# Differential expression of individual transcript variants of PD-1 and PD-L2 genes on Th-1/Th-2 status is guaranteed for prognosis prediction in PCNSL

**DOI:** 10.1038/s41598-019-46473-5

**Published:** 2019-07-10

**Authors:** Yasuo Takashima, Atsushi Kawaguchi, Ryuichi Sato, Kenichi Yoshida, Azusa Hayano, Jumpei Homma, Junya Fukai, Yasuo Iwadate, Koji Kajiwara, Shin Ishizawa, Hiroaki Hondoh, Masakazu Nakano, Seishi Ogawa, Kei Tashiro, Ryuya Yamanaka

**Affiliations:** 1 Laboratory of Molecular Target Therapy for Cancer, Graduate School of Medical Science, Kyoto Prefectural University of Medicine, Kyoto, Japan; 2 Center for Comprehensive Community Medicine, Faculty of Medicine, Saga University, Saga, Japan; 3 Department of Genomic Medical Sciences, Graduate School of Medical Science, Kyoto Prefectural University of Medicine, Kyoto, Japan; 4 Department of Pathology and Tumor Biology, Graduate School of Medicine, Kyoto University, Kyoto, Japan; 5 Department of Neurosurgery, Toyama Prefectural Central Hospital, Toyama, Japan; 6 Department of Neurological Surgery, Wakayama Medical University School of Medicine, Wakayama, Japan; 7 Department of Neurosurgery, Graduate School of Medical Sciences, Chiba University, Chiba, Japan; 8 Department of Neurosurgery, Graduate School of Medical Sciences, Yamaguchi University, Ube, Yamaguchi Japan; 9 Department of Pathology, Toyama Prefectural Central Hospital, Toyama, Japan

**Keywords:** CNS cancer, Prognostic markers, Risk factors, Statistics

## Abstract

In current molecular medicine, next-generation sequencing (NGS) for transcript variant detection and multivariable analyses are valid methods for evaluating gene expression, cancer mechanisms, and prognoses of patients. We conducted RNA-sequencing on samples from patients with primary central nervous system lymphoma (PCNSL) using NGS and performed multivariable analysis on gene expression data and correlations focused on Th-1/Th-2 helper T cell balance and immune checkpoint to identify diagnosis/prognosis markers and cancer immune pathways in PCNSL. We selected 84 transcript variants to limit the analysis range for Th-1/Th-2 balance and stimulatory and inhibitory checkpoints in 31 PCNSLs. Of these, 21 highly-expressed transcript variants were composed of the formulas for prognoses based on Th-1/Th-2 status and checkpoint activities. Using formulas, Th-1^low^, Th-2^high^, and stimulatory checkpoint^high^ resulted in poor prognoses. Further, Th-1^high^Th-2^low^ was associated with good prognoses. On the other hand, CD40-001^high^ and CD70-001^high^ as stimulatory genes, and LAG3-001^high^, PDCD1 (PD-1)-001/002/003^high^, and PDCD1LG2 (PD-L2)-201^low^ as inhibitory genes were associated with poor prognoses. Interestingly, Th-1^high^Th-2^low^ and Th-1^low^Th-2^high^ were correlated with stimulatory checkpoint^low^ as CD70-001^low^ and inhibitory checkpoint^low^ as HAVCR2 (TIM-3)-001^low^ and PDCD1LG2-001/201^low^, respectively. Focused on the inhibitory checkpoint, specific variants of CD274 (PD-L1)-001 and PDCD1-002 served severe hazard ratios. In particular, PDCD1-002^high^ by a cut off score was associated with poor prognoses, in addition to PDCD1-001/003^high^, PDCD1LG2-201^low^, and LAG3-001^high^. These results mainly suggest that expression of transcript variants of PDCD1 and PDCD1LG2 on the Th-1/Th-2 balance enable prognostic prediction in PCNSL. This study provides insights for development of molecular target therapies and identification of diagnosis/prognosis markers in PCNSL.

## Introduction

Primary central nervous system (CNS) lymphomas (PCNSLs) are extranodal non-Hodgkin’s lymphomas (NHLs) of diffuse large B-cell lymphomas (DLBCLs), localized to the brain, eye, meanings, and spinal cord, which are distinct from systemic lymphomas^1,2^. PCNSLs account for approximately 3% of primary CNS tumors and approximately 1% of NHLs in adults^3^. Most PCNSLs are immune-privileged site-associated DLBCLs, according to the World Health Organization (WHO) diagnostic criteria^1,2^. Despite intensive treatments, including high-dose methotrexate (HD-MTX)-based polychemotherapy and deferred whole brain radiotherapy, the median overall survival (OS) time of PCNSLs was associated with poor prognoses (approximately 4 years) compared to extracerebral DLBCLs^4^.

Cancer immunotherapy has advanced by targeting antigens on cell surfaces, as immune checkpoint molecules, which repress killer T cells and pro-inflammatory lymphocytes^5^. Checkpoint inhibitors as monoclonal antibodies block inhibitory checkpoint antigens and repress stimulation of T cells, showing the effects of anticancer activities^6^. The monoclonal antibodies against programmed death 1 (PD-1), also known as cluster of differentiation (CD) 279, and cytotoxic T-lymphocyte-associated protein 4 (CTLA-4; also known as CD152), suppress T-cell receptor (TCR) responses of NHLs^7–11^. In particular, PD-1 blockade with nivolumab is effective in relapse and/or refractory PCNSLs^12,13^. Recent studies have shown that the signal transducer and activator of transcription 3 (STAT3) inhibitors abrogate the expression of PD-1 ligand 1 (PD-L1; also known as CD274), and PD-1 ligand 2 (PD-L2; also known as PDCD1LG2 or CD273) on a lymphoma cell line, HKBML, in addition to an adult T-cell leukemia-lymphoma cell line, ATL-T, and a splenic lymphoma with villous lymphocyte cell line, SLVL^14^. Stimulus-dependent expression of PD-L1 and indoleamine 2,3-dioxygenase 1 (IDO-1) by macrophage-interaction causes immune evasion of PCNSL-derived cell lines HKBML and TK^15^. Besides, a clinicopathological study on 64 PCNSL patients shows that the PD-L1 protein is detected in tumor microenvironments than in tumor cells and is correlated with expression of interferon-gamma (IFN-γ) and CD4 with OS^16^.

Despite various studies and the aforementioned molecular evidences, there are only a few diagnostic and/or prognostic marker candidates in PCNSL. Recently, clinical next-generation sequencing (NGS) enabled an ultra-high-throughput screening for whole genome expression, copy number variation (CNV), single nucleotide variant (SNV) detection in the complete exon, and gene fusion for onco-driver mutation^17–22^. In this study, we conducted high-throughput RNA-sequencing using NGS on tumor tissues from 31 patients with PCNSL, and performed multivariable analysis for their expression and correlations to prognoses, focused on the balance of Th-1 and Th-2 helper T-cell differentiation and expression of immune checkpoint genes to investigate diagnostic and/or prognostic marker candidates and immune checkpoint blockade pathways against CNS lymphomas. We analyzed 84 selected transcript variants derived from 62 genes. Multivariable analysis on the expression analysis composed of the formulas of prognostic prediction and revealed the correlation between the calculated scores of T-cell differentiation status and expression of checkpoint genes, which was associated with prognoses of PCNSL patients.

## Results

### Patient characteristics

This study was performed on specimens from 31 patients with PCNSL whose characteristics were described in Table 1. The median age of the patients was 67 years (range, 31–85 years). Of the 31 patients, 16 patients were female (51.61%), and 15 patients were male (48.38%). The median OS time was 765 days (range, 188–3611 days) (Suppl. Fig. S1A), and the OS was “deceased” in 19 (61.29%) and “living” in 12 patients (38.70%). Univariable and multivariable analyses for OS in gender, age, Karnofsky Performance Status (KPS), Memorial Sloan Kettering Cancer Center (MSKCC) risk score, International Extranodal Lymphoma Study Group (IELSG) risk score, and chemotherapies including ionizing radiation (IR), polychemotherapy, and high dose-methotrexate (HD-MTX), were performed; however, the results did not show any statistically significant difference, except for HD-MTX in univariable analysis (hazard ratio (HR) = 0.2098, 95% confidence interval (CI): 0.0571–0.989, *p* = 0.0486) (Table 1, Suppl. Fig. S1B–G).

**Table 1:** Characteristics of the PCNSL patients examined in this study.

Characteristics	N (%)	OS^a^ (days)				
		Median (Min–Max)	Univariable		Multivariable	
			HR^b^ (95% CI^c^)	P-value	HR (95% CI)	P-value
Total	31 (100)	765 (188-3611)				
Gender						
Male	16 (51.61)	990 (169.8–6378)	1	NA^h^	1	NA
Female	15 (48.38)	951 (273–3738)	0.453 (0.116–1.528)	0.203	0.592 (0.190–1.745)	0.592
Age: Median (Min–Max) 67 (31–85)						
Age < 60	12 (38.70)	994.5 (317.1–6378)	1	NA	1	NA
Age > 60	19 (61.29)	936.9 (169.8–3738)	1.232 (0.494–3.317)	0.658	0.790 (0.181–3.202)	0.738
KPS^d^: Median (Min–Max) 60 (40–90)						
0–60	19 (61.29)	936.9 (169.8–317.1)	1	NA	1	NA
70–100	12 (38.70)	1080 (317.1–6378)	0.426 (0.133–1.160)	0.097	0.241 (0.008–6.561)	0.3491
MSKCC^e^						
1 (Age < 50)	4 (14.81)	1515 (840–3330)	1	NA	1	NA
2 (Age > 50, KPS > 70)	9 (29.03)	951 (317.1–6378)	1.1920 (0.254–8.360)	0.832	2.0524 (0.207–48.228)	0.559
3 (Age > 50, KPS < 70)	18 (58.06)	948.45 (169.8–3171)	2.2253 (0.590–14.563)	0.261	0.9349 (0.109–20.664)	0.956
IELSG^f^						
0-1	6 (19.35)	2175 (317.1–6378)	1	NA	1	NA
2-3	20 (64.51)	807.9 (169.8–3525)	2.7591 (0.819–12.820)	0.106	3.4288 (0.639–24.152)	0.155
4-5	5 (16.12)	1429.2 (936.9–3171)	1.5136 (0.269–8.619)	0.623	0.8690 (0.077–11.912)	0.910
Chemotherapy						
Ionizing radiation	3 (9.67)	762 (169.8–775.8)	1	NA	1	NA
Polychemotherapy	9 (29.03)	1429.2 (317.1–6378)	0.242 (0.054–1.251)	0.085	0.495 (0.046–4.339)	0.528
HD-MTX^g^	19 (61.29)	969 (273–3525)	0.209 (0.057–0.989)	0.048*	0.244 (0.044–1.433)	0.113

Note: ^a^OS; overall survival, ^b^HR; hazard ratio, ^c^CI; confidence interval, ^d^KPS; Karnofsky Performance score, ^e^MSKCC; Memorial Sloan Kettering Cancer Center risk score, ^f^IELSG; International Extranodal Lymphoma Study Group risk score, ^g^HD-MTX; high-dose methotrexate; ^h^NA; not applicable, *P < 0.05, statistically significant.

### Expression patterns of the transcript variants of the genes of interests in PCNSL

**Figure 1:**
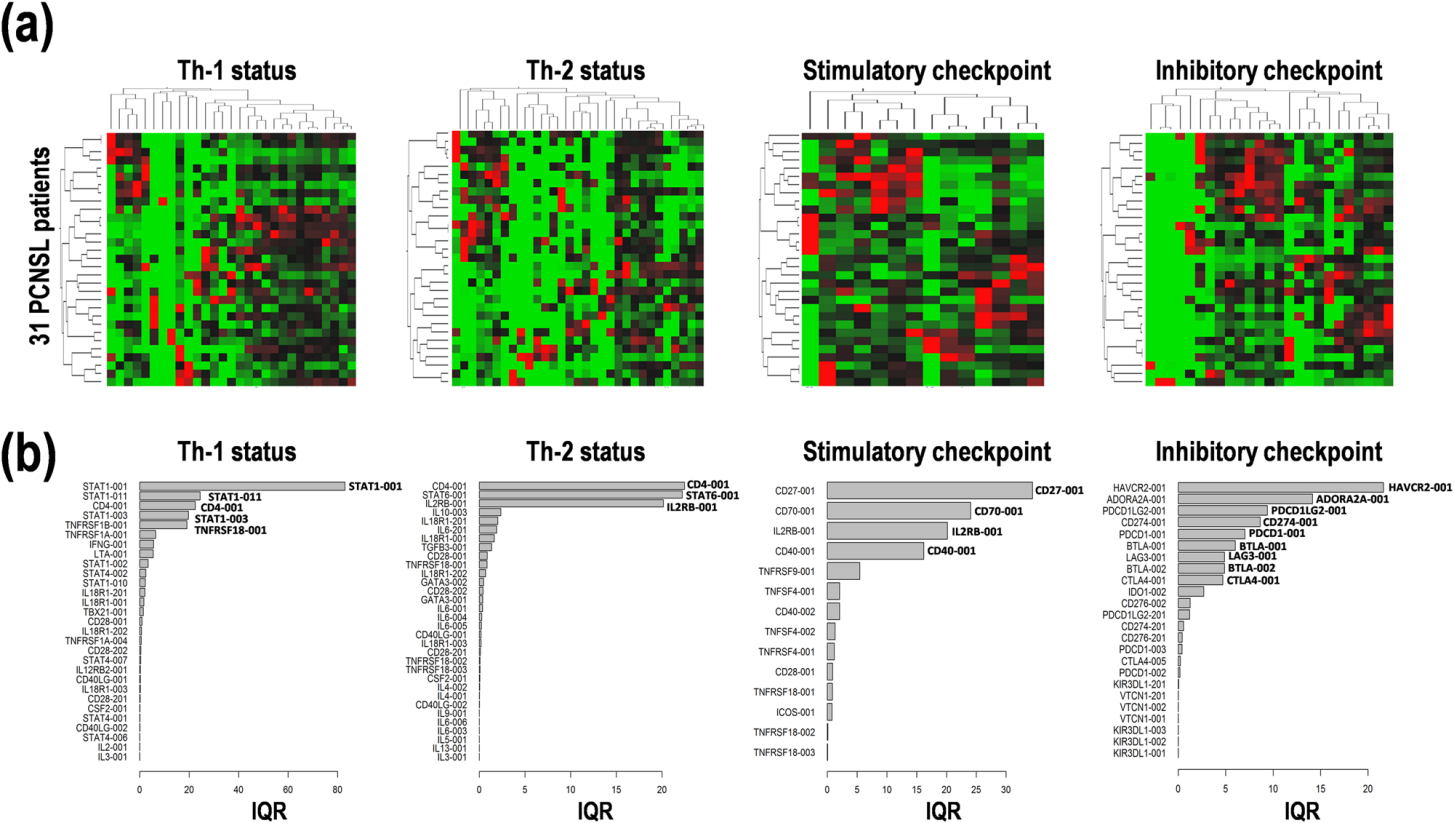
Expression patterns of transcript variants of the genes related to T helper cells type 1/2 (Th-1/Th-2) and immune checkpoint in primary central nervous system lymphoma (PCNSL). (**a**) Hierarchical clustering of relative expression among samples and (**b**) interquartile range (IQR) of the transcript variants of genes related to Th-1, Th-2, stimulatory checkpoint, and inhibitory checkpoint in 31 PCNSL patients. High and low expression is indicated by red and green, respectively, in heat map.

First, to examine the expression of transcript variants of the genes of interests in the 31 PCNSL specimens, we performed NGS using the Illumina HiSeq2000/2500 as a high throughput comprehensive RNA-sequencing for whole transcript variant detection. Recently, cancer immunotherapies have dramatically been improved by the advanced profiling of immune cells and immune checkpoint molecules^5–11^. Therefore, in this study, we focused on cancer immunotherapy-related genes, especially, immune checkpoint genes and genes related to Th-1 and Th-2 differentiation. The expression values of 84 transcript variants derived from a total of 62 selected genes were used for the following multivariable analysis for diagnosis and/or prognosis marker prediction in PCNSL (Suppl. Table S1, Suppl. Fig. S2). Expression data are summarized in the heat map with hierarchical clustering for Th-1 and Th-2 differentiation and stimulatory and inhibitory immune checkpoints (Fig. 1a). In particular, highly interquartile ranges (IQRs) of the representative genes in each were: (i) STAT1-001/003/011, CD4-001, and TNFRSF1B-001 for Th-1 differentiation, (ii) CD4-001, STAT6-001, and IL2RB-001 for Th-2 differentiation, (iii) CD27-001, CD70-001, IL2RB-001, and CD40-001 for stimulatory checkpoint, and (iv) HAVCR2-001, ADORA2A-001, PDCD1LG2-001, CD274-001, PDCD1-001, BTLA-001/002, LAG3-001, and CTLA4-001 for inhibitory checkpoint (Fig. 1b). These data clearly indicate that the specific transcript variants are highly expressed in PCNSL, but not always are all variants expressed.

### Constitution of the prognosis prediction formulas in PCNSL

**Figure 2:**
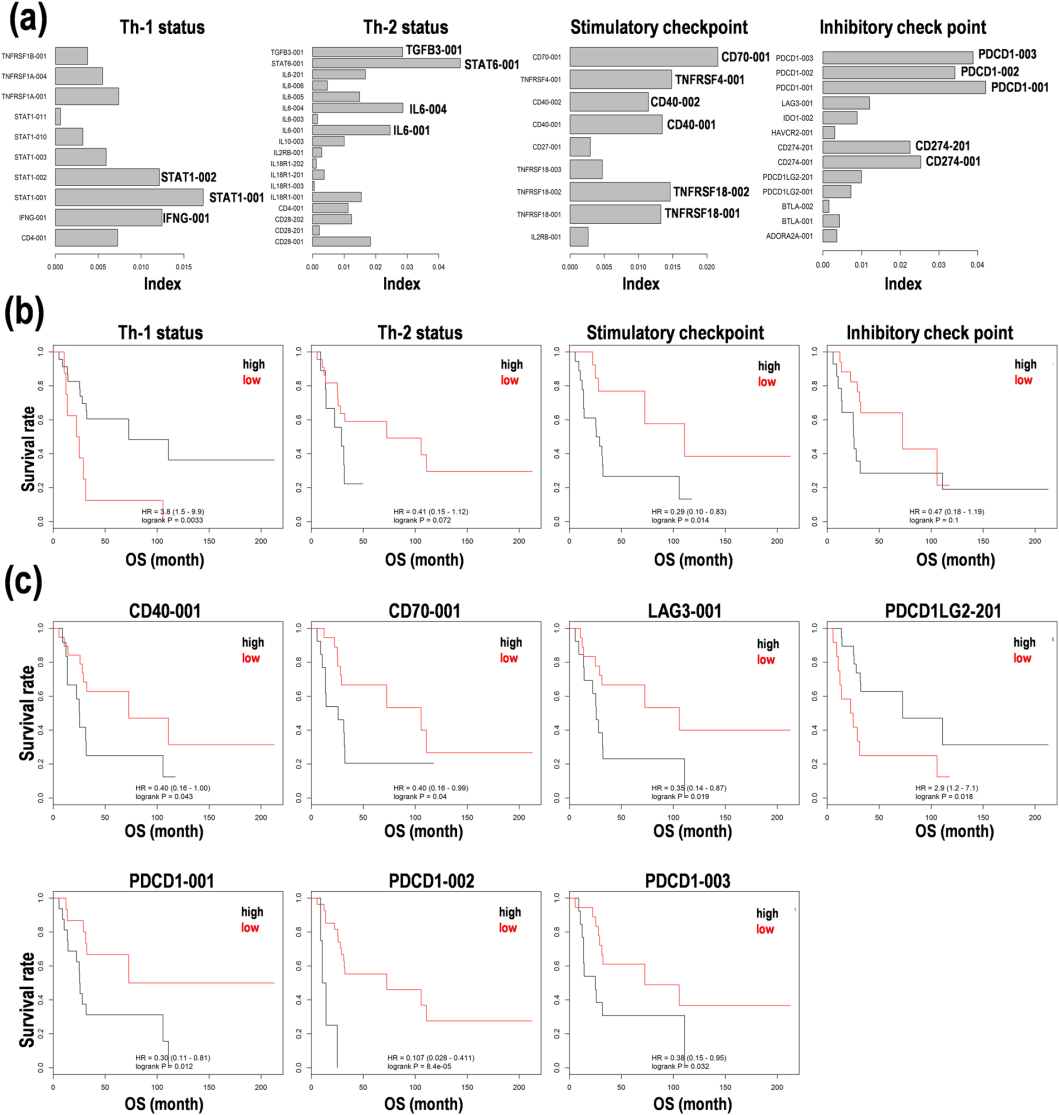
Survival prediction based on T helper cell type 1/2 (Th-1/Th-2) status and immune checkpoint activity in primary central nervous system lymphoma (PCNSL). (**a**) Index calculated from combined methods of random survival forests analysis and principal component analysis (PCA). (**b**) Kaplan-Meier analysis on the survival prediction formula in 31 patients with PCNSL. The patients were divided into two subgroups with high (black line) and low (red line) scores from the median score calculated based on the formula. (**c**) Kaplan-Meier analysis of the expression levels of representative genes for immune checkpoint. CD40 and CD70 as stimulatory checkpoint genes. Lymphocyte activation gene 3 (LAG3), programmed cell death ligand 2 (PDCD1LG2), and programmed cell death 1 (PDCD1) as inhibitory checkpoint genes. The patients were divided into two subgroups with high (black line) and low (red line) expression by the median expression of the gene. Hazard ratio (HR) with 95% confidence interval (CI) and *p*-value from log rank test were calculated. OS; overall survival.

Second, based on the IQRs in each category, we calculated the index contributing to Th-1 and Th-2 differentiation and immune checkpoints on the clinical information as OS times using Cox regression model, random forests analysis, and principal component analysis (PCA) (Fig. 2a). In particular, STAT1-001, STAT6-001, CD40-001, CD70-001, CD274-001, and PDCD1-001 possessing high index were also highly expressed in PCNSL (Fig. 1b). The index was normalized using standard deviations of variables and used to generate formulas, as the sum of the integration of the coefficients calculated from Cox regression analyses and the fragments per kilobase of exon per million mapped fragments (FPKM) values of genes, to estimate the status of patients with PCNSL like prognoses, as follows:

Th-1 Status = 0.007 × CD4-001 + 0.012 × IFNG-001 + 0.017 × STAT1-001 + 0.012 × STAT1-002 + 0.006 × STAT1-003 + 0.003 × STAT1-010 + 0.001 × STAT1-011 + 0.007 × TNFRSF1A-001 + 0.006 × TNFRSF1A-004 + 0.004 × TNFRSF1B-001

Th-2 Status = 0.018 × CD28-001 + 0.002 × CD28-201 + 0.012 × CD28-202 + 0.011 × CD4-001 + 0.015 × IL18R1-001 + 0.001 × IL18R1-003 + 0.004 × IL18R1-201 + 0.001 × IL18R1-202 + 0.003 × IL2RB-001 + 0.01 × IL10-003 + 0.025 × IL6-001 + 0.002 × IL6-003 + 0.029 × IL6-004 + 0.015 × IL6-005 + 0.005 × IL6-006 + 0.017 × IL6-201 + 0.047 × STAT6-001 + 0.029 × TGFB3-001

Stimulatory checkpoint = 0.003 × IL2RB-001 + 0.013 × TNFRSF18-001 + 0.015 × TNFRSF18-002 +0.005 × TNFRSF18-003 + 0.003 × CD27-001 + 0.013 × CD40-001 + 0.011 × CD40-002 + 0.015 ×TNFRSF4-001 + 0.022 × CD70-001

Inhibitory checkpoint = 0.004 × ADORA2A-001 + 0.004 × BTLA-001 + 0.002 × BTLA-002 + 0.007 × PDCD1LG2-001 + 0.01 × PDCD1LG2-201 + 0.025 × CD274-001 + 0.023 × CD274-201 + 0.003 × HAVCR2-001 + 0.009 × IDO1-002 + 0.012 × LAG3-001 + 0.042 × PDCD1-001 + 0.034 × PDCD1-002 + 0.039 × PDCD1-003

### Candidates for the prognosis markers in PCNSL

The subgroups by the median scores calculated by each formula divided Kaplan-Meier curves (Fig. 2b). In particular, the subgroups with Th-1^low^ (hazard ratio (HR) = 3.8, 95% confidence interval (CI): 1.5–9.9, *p* = 0.0033) and stimulatory checkpoint^high^ (HR = 3.4, 95%CI: 1.2–10.0, *p* = 0.014) were associated with poor prognoses with statistical differences (Fig. 2b). Th-2^high^ (HR = 2.4, 95%CI: 0.8–6.6, *p* = 0.072) and inhibitory checkpoint^high^ (HR = 2.1, 95%CI: 0.8–5.6, *p* = 0.1) also correlated with poor prognoses (Fig. 2b). As for representative marker candidates of stimulatory checkpoint genes, CD40-001^high^ (HR = 2.5, 95%CI: 1.0–6.2, *p* = 0.043) and CD70-001^high^ (HR = 2.5, 95%CI: 1.0–6.2, *p* = 0.04) were associated with poor prognoses (Fig. 2c). On the other hand, as for inhibitory checkpoint genes, LAG3-001^high^ (HR = 2.8, 95%CI: 1.1–7.1, *p* = 0.019), PDCD1LG2-201^low^ (HR = 2.9, 95%CI: 1.2–7.1, *p* = 0.018), PDCD1-001^high^ (HR = 3.3, 95%CI: 1.2–9.1, *p* = 0.012), PDCD1-002^high^ (HR = 9.3, 95%CI: 2.4–35.7, *p* = 8.4E-05), and PDCD1-003^high^ (HR = 2.6, 95%CI: 1.1–6.7, *p* = 0.032) were associated with poor prognoses (Fig. 2c). Besides, the subgroups with IL2RB-001^high^, TNFRSF18-001^high^, TNFRSF18-002^high^, CD27-001^high^, CD40-002^high^, TNFRSF4-001^high^ (Suppl. Fig. S3A), and TNFRSF18-003^low^ (Suppl. Fig. S3B) were associated with poor prognoses, but were not significantly different, as for stimulatory checkpoint genes. Similarly, ADORA2A-001^high^, PDCD1LG2-001^high^, HAVCR2-001^high^ (Suppl. Fig. S4A), BTLA-001/002^low^, CD274-001/201^low^, and IDO1-002^low^ (Suppl. Fig. S4B) were associated with poor prognoses, but were not statistically significant, as for inhibitory checkpoint genes. The specific transcript variants such as CD40-001, CD70-001, LAG3-001, PDCD1LG2-201, and PDCD1-001/002/003 are candidates for immune checkpoint genes for promising prognosis factors to predict OS of PCNSL patients.

### Assessment of the balance of Th-1 and Th-2 differentiation in PCNSL

**Figure 3:**
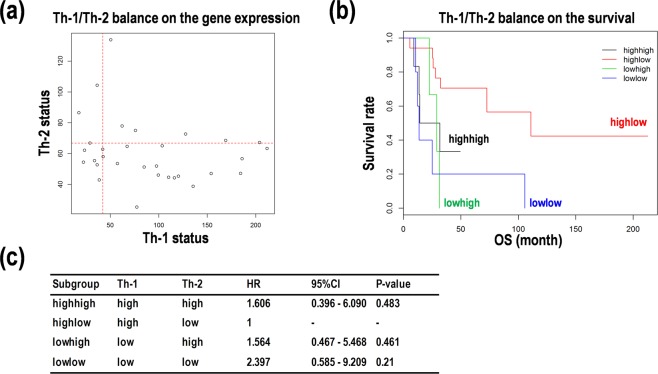
Balance of T helper cell type 1/2 (Th-1/Th-2) predicts prognoses in primary central nervous system lymphoma (PCNSL). (**a**) The balance of Th-1 and Th-2 status in scatter plot. (**b**) Kaplan-Meier analysis on the survival prediction formula in PCNSL patients. The patients were divided into four subgroups with Th-1^high^Th-2^high^ (black), Th-1^high^Th-2^low^ (red), Th-1^low^Th-2^high^ (green), and Th-1^low^Th-2^low^ (blue) by the median score calculated on the formula. OS; overall survival. (**c**) Comparison of risk in survival of PCNSL with Th-1/Th-2 balance. Hazard ratio (HR) with 95% confidence interval (CI) compared with the Th1^high^Th2^low^ subgroup.

We next wanted to identify facilitating factors to divide Kaplan-Meier curves and/or to enlarge HR values in the survival analysis in PCNSL. We focused on the balance in Th-1 and Th-2 differentiation. As shown in Fig. 3a, the calculated Th-1 scores were distributed at a wide range, but the calculated Th-2 scores were compacted. The four subgroups with Th-1^high^Th-2^high^, Th-1^high^Th-2^low^, Th-1^low^Th-2^high^, and Th-1^low^Th-2^low^ were generated. Except for Th-1^high^Th-2^low^, the other three subgroups were associated with poor prognoses in the Kaplan-Meier curves (Fig. 3b,c). While, Th-1^low^Th-2^low^ was associated with the worst prognosis among the four subgroups (HR = 2.4, 95% CI: 0.6-9.2, *p* = 0.21) (Fig. 3b,c). These results suggest that the Th-1 activity and the Th-2 inactivity would contribute to prolonged OS of the PCNSL patients.

### Overlay of the transcript variant expression on the Th-1/Th-2 balance in PCNSL

**Figure 4:**
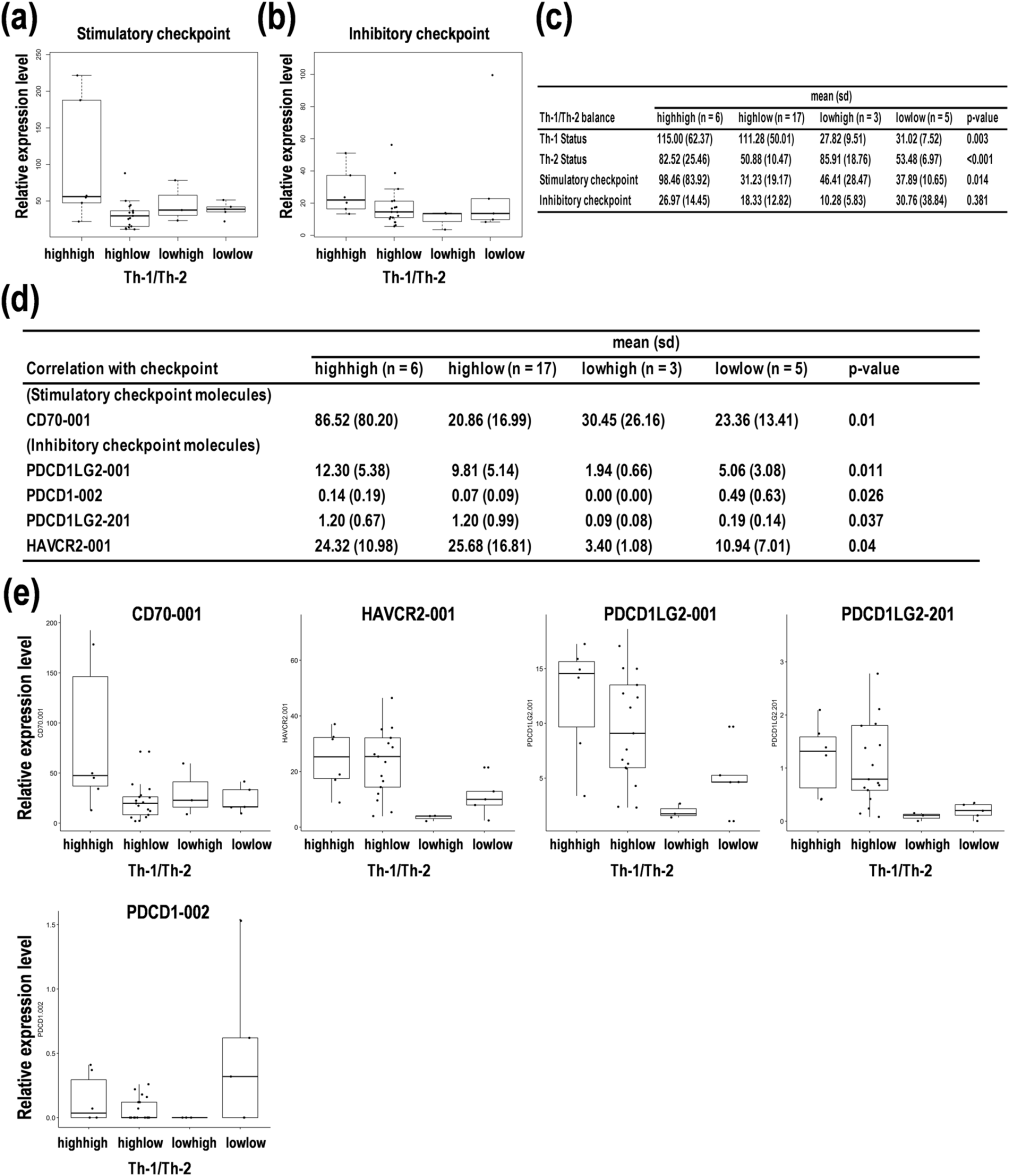
Comparative expression analysis of immune checkpoint-related genes on the balance of T helper cell type 1/2 (Th-1/Th-2) status in primary central nervous system lymphoma (PCNSL). (**a**,**b**) Relative expression patterns of immune checkpoint-related genes based on the balance of Th-1/Th-2 status. (**a**) Stimulatory checkpoint. (**b**) Inhibitory checkpoint. (**c**) Statistics for the four subgroups defined as Th-1^high^Th-2^high^, Th-1^high^Th-2^low^, Th-1^low^Th-2^high^, and Th-1^low^Th-2^low^. The p-value indicates one-way analysis of variance (ANOVA). (**d**) Statistics for the differential expression of the genes on the four groups. The p-value indicates one-way ANOVA. (**e**) The box-whisker plots of the expression of stimulatory and inhibitory immune checkpoint genes. The PCNSL patients were divided into four subgroups including Th-1^high^Th-2^high^, Th-1^high^Th-2^low^, Th-1^low^Th-2^high^, and Th-1^low^Th-2^low^.

The expression patterns of transcript variants of genes of interests were examined to investigate the effects on the Th-1/Th-2 balance. In the balance between Th-1/Th-2 differentiation and stimulatory checkpoint genes, lower and higher expression of stimulatory checkpoint genes were overlaid on the Th-1^high^Th-2^low^ and Th-1^low^Th-2^high^ balances, respectively (Fig. 4a,c), whereas higher and lower expression of inhibitory checkpoint genes was detected on the Th-1^high^Th-2^low^ and Th-1^low^Th-2^high^ balances, respectively (Fig. 4b,c), indicating that reciprocal patterns were found in stimulatory and inhibitory checkpoint genes on the Th-1/Th-2 balance. In addition, the changes in Th-1/Th-2 balance were diffused in stimulatory checkpoint genes (*p* = 0.014) but not in inhibitory checkpoint genes (*p* = 0.381), in addition to the spread changes in Th-1 (*p* = 0.003) and Th-2 scores (*p* < 0.001) (Fig. 4c). Additionally, lower and higher expression of CD70-001 on Th-1^high^Th-2^low^ and Th-1^low^Th-2^high^ were found in the stimulatory checkpoint genes, respectively (Fig. 4d,e). Similarly, CD27-001 and CD40-001/002 showed similar results with no statistical significances (Suppl. Tables S2, S3, and Suppl. Fig. S5). Inversely, higher and lower expression of PDCD1LG2-001/201, PDCD1-002, and HAVCR2-001 on Th-1^high^Th-2^low^ and Th-1^low^Th-2^high^ were found in the inhibitory checkpoint genes, respectively (Fig. 4d,e, and Suppl. Table S2). Similarly, BTLA-001/002, CD274-001/201, IDO1-002, LAG3-001, and PDCD1-001/003 showed similar results with no statistical significances (Suppl. Tables S2, S4, and Suppl. Fig. S6). These results suggest that lower expression of stimulatory checkpoint genes is correlated with the Th-1^high^Th-2^low^ balance, whereas higher expression of inhibitory checkpoint genes is correlated with the Th-1^low^Th-2^high^ balance. Coupled with the aforementioned results in Figs 2–4, these data clearly suggest that higher expression of inhibitory checkpoint genes on the Th-1^low^Th-2^high^ balance is correlated with a poorer prognosis in PCNSL.

### Inhibitory checkpoint genes are satisfied with central factors for prognosis prediction in PCNSL

**Figure 5:**
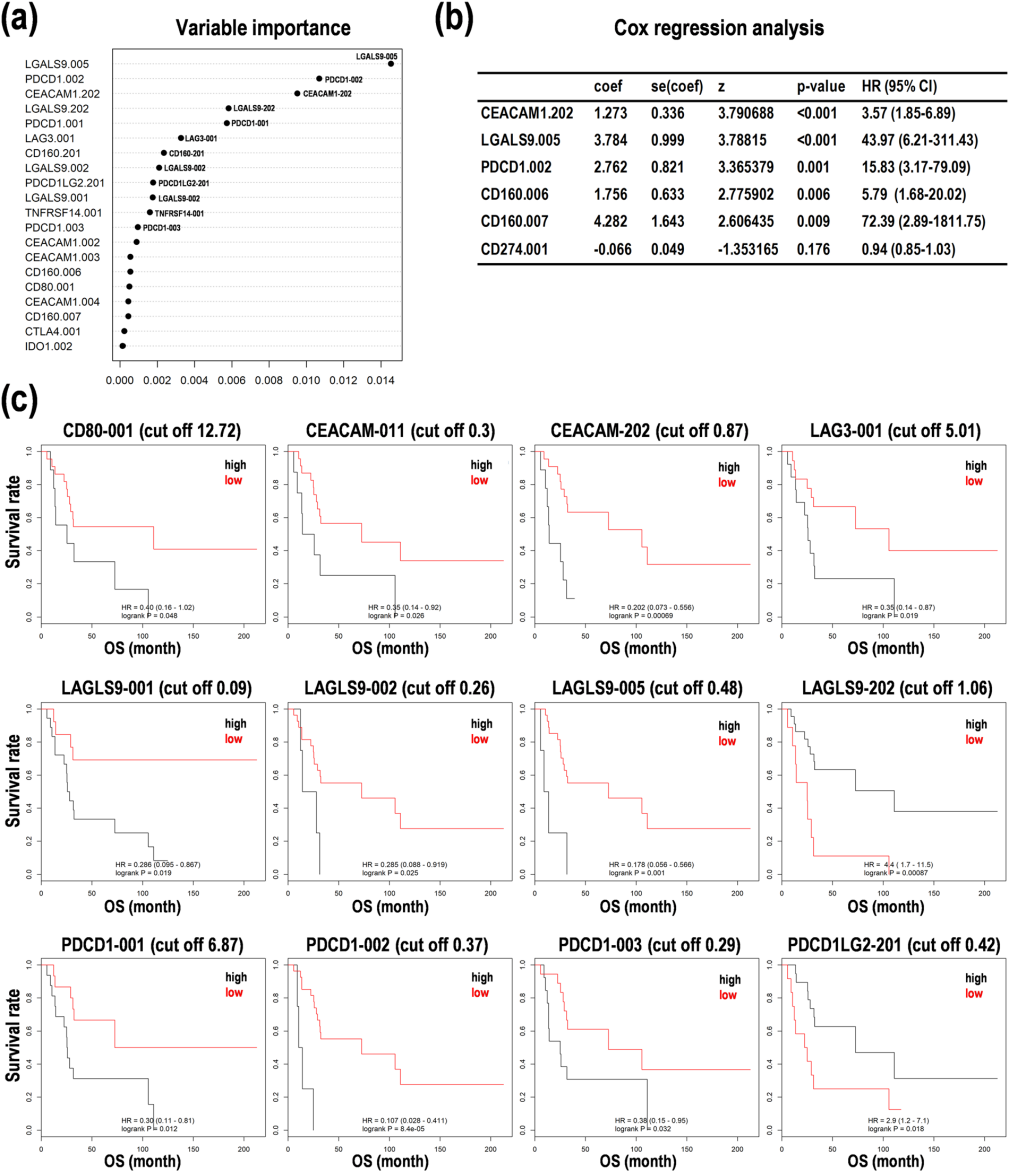
Random forests survival analysis and Cox regression analysis to predict prognoses with the expression of inhibitory checkpoint genes in primary central nervous system lymphoma (PCNSL). (**a**) Variable importance derived from a random forests survival analysis. (**b**) Cox regression analysis for representative genes including programmed cell death 1 (PDCD1), CD274 (PD-L1), CD160, LGALS9, and CEACAM1. Akaike information criterion (AIC)-based optimization was performed, and hazard ratios with 95% confidence interval (CI) were shown. (**c**) Kaplan-Meier analysis on the expression levels of representative genes for inhibitory checkpoint. The patients were divided into the two subgroups with high (black line) and low (red line) expression by the cutoff score of the expression of the transcript variants, including programmed cell death 1 (PDCD1), programmed cell death ligand 2 (PDCD1LG2) (=PD-L2), CD80, LAG3, LGALS9, and CEACAM. Hazard ratio (HR) with 95% and p-value from log rank test were calculated. OS; overall survival.

Considering the aforementioned results, we next focused on the inhibitory checkpoint genes. After random survival forests analysis and PCA, we found that each variable importance of PDCD1-001/002/003, KIR3DL1-002, PDCD1LG2-201, LAG3-001, and CD274-001 especially contributed to OS of PCNSL patients (Fig. 5a). Cox regression analysis also revealed that higher expression of PDCD1-002 (HR = 15.83, 95%CI: 3.17-79.09, *p* = 0.001), CD160-006 (HR = 5.79, 95%CI: 1.68-20.02, *p* = 0.006), CD160-007 (HR = 72.39, 95%CI: 2.89-1811.75, *p* = 0.009), CEACAM1-202 (HR = 3.57, 95%CI: 1.85-6.89, *p* < 0.001), LGALS9-005 (HR = 43.97, 95%CI: 6.21-311.43, *p* < 0.001), in addition to CD274-001 (HR = 0.94, 95%CI: 0.85-1.03, *p* = 0.176), was correlated with higher hazard ratios for OS (Fig. 5b).

The Kaplan-Meier survival analysis also showed that the identical cut off values on each expression of PDCD1-001 (cut off = 6.87), PDCD1-002 (cut off = 0.37), PDCD1-003 (cut off = 0.29), PDCD1LG2-201 (cut off = 0.42), and LAG3-001 (cut off = 5.01) reconstituted from the Kaplan-Meier results by their median expression (Figs 2c and 5c), in addition to higher expression of CD80-001 (cut off = 12.72), CEACAM1-011 (cut off = 0.3), CEACAM1-202 (cut off = 0.87), LGALS9-001 (cut off = 0.09), LGALS9-002 (cut off = 0.26), and LGALS9-005 (cut off = 0.48) with poor prognoses (Fig. 5c). On the other hand, higher expression of CD160-006/007/202, CD276-002/201, CD86-002, CD96-001, CEACAM1-003/004/005, CTLA4-001/005, HAVCR2-201, LGALS3-001, PDCD1LG2-001, PVR-002/003/006, TIGIT-201, TMIGD2-001, TNFRSF14-001/009, KIR3DL1-201, and VTCN1-201 by each cut off value correlated with poorer prognoses with no statistical significance in PCNSL (Suppl. Fig. S7). Inversely, lower expression of LGALS9-002 (cut off = 1.06) was associated with poorer prognoses (Fig. 5c). In addition, lower expression of BTLA-001/002, C10orf54-001, CD160-201, CD274-001/201, CD80-202, CD86-001/201, CD96-002, CEACAM1-001/002, IDO1-002, LGALS9-201, PVR-004, KIR3DL1-003, and VTCN1-001/002 by each cut off value was associated with poor prognoses with no statistically significant in PCNSL (Suppl. Fig. S8). These results suggest that the specific transcript variants derived from inhibitory checkpoint genes, especially PDCD1-001/002/003, PDCD1LG2-201, and LAG3-001, would be satisfied with central factor candidates for prognosis prediction in PCNSL. In other word, these transcript variants may be promising prognosis marker candidates in PCNSL.

### Correlation analysis among Th-1/Th-2 differentiation and immune checkpoint genes in PCNSL

**Figure 6:**
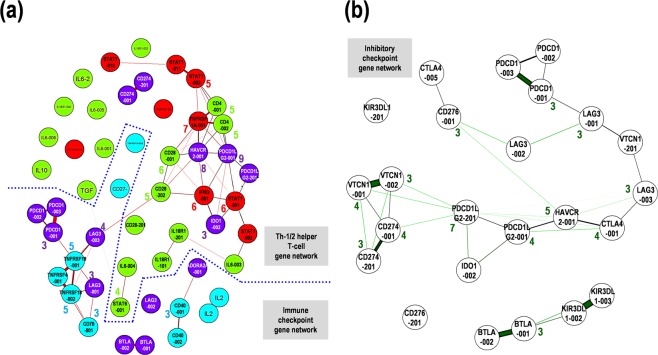
Schematic representation of the correlation between the gene expression in the T helper cell type 1/2 (Th-1/Th-2) status and immune checkpoint in primary central nervous system lymphoma (PCNSL). (**a**) Correlation among Th-1 (red) and Th-2 (green), stimulatory checkpoint (blue), and inhibitory checkpoint (purple). (**b**) Correlation among inhibitory checkpoint molecules. Thick and thin lines with a distance indicate strong and weak correlation between the expression levels of the two genes. The numbers with circles indicate the numbers of nodes over two.

To validate the correlation between expression of checkpoint genes and the Th-1 and Th-2 status, additional analysis for the correlation among Th-1/Th-2 differentiation and expression of stimulatory and inhibitory immune checkpoint genes was carried out. The analysis for the correlations between multiple pairs of variables returned representative Pearson’s rank correlation coefficient values (r) with statistical significances by additional nonparametric analyses, which were summarized in the matrix (Suppl. Fig. S9A). The variable 1, including HAVCR2-001, LAG3-001, PDCD1LG2-001, ICOS-001, IDO1-002, and CTLA4-001, is correlated with the variable 2, including CD28-001, STAT4-002, IFNG-001, CD4-001, CD28-202, and TBX21-001, with relative high correlation coefficient values (r > 0.47, *p* < 0.05) (Suppl. Fig. S9A). The results suggested that a complex correlation network was constituted of the variable 1, mainly composed of inhibitory checkpoint genes, and the variable 2, principally composed of developmental status of Th-1 differentiation (Suppl. Fig. S9A). Nonparametric analyses with Spearman, Kendall rank distance, and Hoeffding independence test also indicated that the variable 1, including BTLA-002, CD274-001, HAVCR2-001, ICOS-001, LAG3-001, PDCD1LG2-001/201, STAT4-002, TNFRSF18-001, and TNFRSF1A-001, was correlated with the variable 2, including BTLA-001, PDCD1LG2-001, TNFRSF1A-001, CD28-001, TBX21-001, CD4-001, STAT1-001, and IFNG-001, with relative high correlations (r > 0.3, *p* < 0.05) (Suppl. Fig. S9B). Besides, in part of the genes analyzed, the schematic representation of their correlation with graphical lasso showed that HAVCR2-001 and PDCD1LG2-001, both inhibitory checkpoint genes, were pivotal factors with important nodes in the Th-1/Th-2 network, followed by TNFRSF1A-001, IFNG-001, STAT1-001, and CD4-001 (Fig. 6a). CD28-202-to-LAG3-003 interaction connected the Th-1/Th-2 gene network and the immune checkpoint gene network, suggestive of an important network hub between the two (Fig. 6a). Further, focused on the inhibitory checkpoint network and extracted them, PDCD1LG2-201, CD274-001, and VTCN1-001/002 seemed a network hub into the complex inhibitory checkpoint gene network (Fig. 6b). These correlation analysis results suggest that expression control of the hub genes with several nodes, including HAVCR2-001, PDCD1LG2-001/201, CD274-001, and VTCN1-001/002, can reconstitute the complex network composed of Th-1/Th-2 status and immune checkpoint genes and their balances.

### Pathway analysis on the cancer immunotherapy-related genes in PCNSL

We finally performed gene set enrichment analysis (GSEA) using the dataset. In this study, 7565 known genes were detected after sequencing, and the 337 genes of these found in the Kyoto Encyclopedia of Genes and Genomes (KEGG) database. While, the genes with differential expression in the PCNSL subgroup with poor prognoses (cutoff by median OS), compared that with good prognoses, were 3140, and the 59 genes found in the KEGG. Of these, the average expression of the two genes, including CD70 and PDCD1, and the 12 isoforms derived from the seven genes, including CD40, CD70, IL6, IL10, STAT1, STAT6, and TNFRSF14, were cancer immunotherapy-related genes (false discovery rate (FDR) < 0.01) (Table 2). In the GSEA with KEGG, 10 pathways on “expression analysis of the gene” and 16 pathways on “expression analysis of the transcript variant” analysis included 31 genes and 139 transcript variants, respectively (*p* < 0.05; Table 3, and FDR < 0.01; Suppl. Table S5). In particular, T cell receptor signaling pathway (KEGG ID: hsa04660) (Suppl. Fig. S10), cytokine-cytokine receptor interaction (hsa04060) (Suppl. Fig. S11), and cell adhesion molecules (hsa04514) (Suppl. Fig. S12) may be involved in cancer immunotherapy. While, since the complete data of all genes or transcripts is more unbiased in the PCNSL clinical samples, systematic approaches may return different results.

**Table 2:** Differential expression of genes in the poor prognosis subgroup, compared to the good prognosis subgroup.

Symbol	Transcript ID	logFC^a^	logCPM^b^	LR^c^	P Value	FDR^d^
(Gene)						
CD70	ENSG00000125726	1.168	5.469	32.985	9.28E-09	1.26E-06
PDCD1	ENSG00000188389	1.301	3.962	29.381	5.94E-08	7.07E-06
(Isoform)						
CD70	ENST00000245903	1.0515	5.329	132.250	1.32E-30	2.83E-28
STAT1	ENST00000361099	−0.622	6.567	121.864	2.47E-28	4.89E-26
IL10	ENST00000423557	−0.565	5.661	52.857	3.59E-13	3.14E-11
STAT6	ENST00000555375	1.048	3.227	25.665	4.06E-07	1.68E-05
STAT6	ENST00000557781	0.884	3.590	25.354	4.77E-07	1.95E-05
STAT1	ENST00000409465	0.593	4.532	24.688	6.74E-07	2.70E-05
STAT6	ENST00000554764	1.138	2.699	18.404	1.79E-05	5.36E-04
STAT6	ENST00000555641	0.769	3.468	17.572	2.77E-05	7.96E-04
STAT6	ENST00000555222	0.617	3.759	14.580	1.34E-04	3.27E-03
TNFRSF14	ENST00000451778	2.290	1.562	14.533	1.37E-04	3.35E-03
CD40	ENST00000461171	0.731	3.259	13.363	2.56E-04	5.81E-03
IL6	ENST00000426291	2.267	1.478	12.360	4.38E-04	9.26E-03

Note: ^a^FC; fold change, ^b^CPM; counts per million, ^c^LR; likelihood ratio, and ^d^FDR; false discovery rate. Subgroups were divided by the median overall survival of PCNSL patients analyzed.

**Table 3:** The target pathway candidates in PCNSL.

Pathway	Pathway name	KEGG PathGenes	Chip PathGenes	Target AllGenes	Target PathGenes	P Value
(Gene)						
hsa04024	cAMP signaling pathway - Homo sapiens (human)	212	211	361	24	0.00041
hsa04080	Neuroactive ligand-receptor interaction - Homo sapiens (human)	338	337	361	31	0.00153
hsa05168	Herpes simplex virus 1 infection - Homo sapiens (human)	492	489	361	9	0.00214
hsa04062	Chemokine signaling pathway - Homo sapiens (human)	190	187	361	18	0.0103
hsa05020	Prion diseases - Homo sapiens (human)	35	35	361	6	0.01052
hsa04621	NOD-like receptor signaling pathway - Homo sapiens (human)	178	175	361	2	0.02537
hsa04514	Cell adhesion molecules (CAMs) - Homo sapiens (human)	144	140	361	13	0.03052
hsa05169	Epstein-Barr virus infection - Homo sapiens (human)	201	199	361	3	0.03652
hsa05014	Amyotrophic lateral sclerosis (ALS) - Homo sapiens (human)	51	51	361	6	0.04576
hsa05414	Dilated cardiomyopathy (DCM) - Homo sapiens (human)	91	90	361	9	0.04751
(Isoform)						
hsa04080	Neuroactive ligand-receptor interaction - Homo sapiens (human)	338	337	3140	59	0.00001
hsa04060	Cytokine-cytokine receptor interaction - Homo sapiens (human)	294	293	3140	74	0.00011
hsa05166	Human T-cell leukemia virus 1 infection - Homo sapiens (human)	219	219	3140	139	0.00016
hsa05010	Alzheimer disease - Homo sapiens (human)	171	152	3140	104	0.00017
hsa04218	Cellular senescence - Homo sapiens (human)	160	157	3140	101	0.00089
hsa04110	Cell cycle - Homo sapiens (human)	124	124	3140	82	0.00152
hsa05034	Alcoholism - Homo sapiens (human)	180	180	3140	111	0.00172
hsa04144	Endocytosis - Homo sapiens (human)	244	233	3140	136	0.0025
hsa04932	Non-alcoholic fatty liver disease (NAFLD) - Homo sapiens (human)	149	143	3140	89	0.00355
hsa03050	Proteasome - Homo sapiens (human)	45	44	3140	35	0.00597
hsa05170	Human immunodeficiency virus 1 infection - Homo sapiens (human)	212	205	3140	118	0.00637
hsa04210	Apoptosis - Homo sapiens (human)	136	134	3140	81	0.01005
hsa05211	Renal cell carcinoma - Homo sapiens (human)	69	65	3140	44	0.01483
hsa05167	Kaposi sarcoma-associated herpesvirus infection - Homo sapiens (human)	186	184	3140	103	0.01815
hsa04660	T cell receptor signaling pathway - Homo sapiens (human)	101	99	3140	60	0.02279
hsa05164	Influenza A - Homo sapiens (human)	171	170	3140	92	0.04659

## Discussion

Here, we performed NGS for distinct transcript variant detection and multivariable analyses for evaluating prognoses in 31 patients suffering PCNSL and for discovering a stimulus-dependent oncopathway input from tumor microenvironments, including activated T cells, and stimulatory and inhibitory immune checkpoints in PCNSL. In particular, we focused on the correlation between checkpoint genes and Th-1/Th-2 differentiation to estimate OS of PCNSL patients. Data showed lower and higher expression of Th-1 and Th-2 differentiation genes with poorer prognoses, respectively. CD40-001^high^ and CD70-001^high^ as stimulatory checkpoint genes and LAG3-001^high^, PDCD1 (PD-1)-001/002/003^high^, and PDCD1LG2 (PD-L2)-201^low^ as inhibitory checkpoint genes also were associated with poorer prognoses. Th-1^high^Th-2^low^ and Th-1^low^Th-2^high^ were correlated with lower expression of CD70-001, and PDCD1LG2-001/201 and HAVCR2 (TIM-3)-001 for inhibitory checkpoint. For inhibitory checkpoint genes, Cox regression analysis showed higher HR in the expression of CD274 (PD-L1)-001, CD160-006/007, LGALS9-005, CEACAM1-202, and PDCD1-002. Further, higher expression of inhibitory checkpoint genes, including PDCD1-001/002/003, PDCD1LG2-201, and LAG3-001, with a cut off score reconstituted successfully the Kaplan-Meier curves estimated by the median expression. Besides, correlation coefficient analyses indicated that inhibitory checkpoint genes, including HAVCR2-001 and PDCD1LG2-001, governed the Th-1/Th-2 differentiation network. In addition, CD28-202 genetically interacted with LAG3-001, a hub gene of the checkpoint gene network bridging to the Th-1/Th-2 network in PCNSL. The GSEA with KEGG also clarified gene networks harboring differential expression of the PDCD1 gene, T-cell receptor signaling, cytokine interaction, and cell adhesion. These results suggest that identical expression of transcript variants of inhibitory immune checkpoint genes overlaid on the Th-1/Th-2 balance enables to predict survival distributions in PCNSL patients.

On the other hand, we also examined the dataset of DLBCL (n = 47) deposited in The Cancer Genome Atlas (TCGA) for the Th-1/Th-2 status and the immune checkpoint molecule scores (Suppl. Figs S13 and S14). The results from the DLBCL were as follows: (i) Th-1 score was correlated with Th-2 score (Suppl. Fig. S13E). (ii) Th-1^low^ was correlated with lower expression of inhibitory checkpoint gene expression (Suppl. Fig. S13I). (iii) The LGALS9^low^ showed poor prognoses (Suppl. Fig. S14F). (iv) Differential expression of PDCD1 or PDCD1LG2 did not divide survival curves in DLBCL (Suppl. Fig. S14H and I). Hence, we considered that the correlation between Th-1/Th-2 balance and checkpoint gene expression is significant in PCNSL but not in DLBCL. Thus, this study may provide insights for development of molecular target therapies and identification of diagnosis and prognosis markers based on NGS and multivariable analysis in PCNSL.

As described above, we only examined the Th-1/Th-2 balance and checkpoint genes, including PD-1, the ligands, and the other antigen molecules. However, the differentiation status of other T cells such as regulatory T cells (Treg)^23–28^, Th-17 cells^27^, CD4^+^ ^27^, CD8^+^ cells^16^, and macrophages within tumor microenvironments^15^ contribute to immune checkpoint activity via intrinsic and extrinsic factors in tumor cells or T cells^14,29–31^. On the other hand, MTX is an antifolate that inhibits DNA syntheses^32^ and the expression of glucocorticoid receptors in human blood cells^33^. HD-MTX treatment and deferred radiotherapy are a standard protocol for PCNSL treatment; nevertheless, most of the cases come to relapse-acquired resistances^4^. Recent studies showed that immune checkpoint blockade with monoclonal antibodies against the cell surface antigens, including CTLA-4, ipilimumab, and PD-1, nivolumab and/or pembrolizumab^34^, and a combination anti-PD-1/CTLA-4 regimens (nivolumab- ipilimumab) have been effective against melanoma^35^, lung cancer^31^, gastrointestinal tract cancer^36^, urologic cancer^37^, and liver cancer^38^. However, it has also been reported that tumor and T-cell intrinsic and extrinsic factors contribute to immunotherapy resistances such as adaptive immune and acquired resistances, except for patients who have primary resistance to checkpoint inhibitors^30^. Hence, it is important to prevent the recurrences with chemical and checkpoint inhibition resistance in PCNSL treatments^39,40^.

In addition to CTLA-4 and PD-1, recent trends shifted to alternative inhibitory receptors and their mechanisms within tumor microenvironments. LAG-3 is considered the third inhibitory receptor candidate in clinics in the next generation^41^, whereasTIM-3 is expressed in FoxP3^+^ Treg and activates Treg function, and the TIM-3 blockade has therapeutic effects in a preclinical model^42^. TIM-3 also functions on the IFN-γ-producing T-cells, macrophages, and dendritic cells, where it leads to the inhibition of Th-1 responses^42^. Therefore, multi-targeting of LAG-3, TIM-3, PD-1, and/or CTLA-4 may serve as a next generation cancer immunotherapy. However, these molecules are also responsible for a primary or adaptive resistance for immunotherapy^30^. PDCD1LG2 also functions in the PD-1 blockade such as on PD-L1, showing a potential resistance mechanism in immune checkpoint inhibition^30^. Hence, these inhibitory checkpoint genes may also be difficult to assign target molecules in part of PCNSLs. This study identified promising diagnosis and/or prognosis marker candidates and potential target genes as hub genes (i.e., PDCD1LG2-001/201, HAVCR2-001, CD274-001, VITCN1-001/002, CD28-202, and LAG3-001/003) connecting the Th-1/Th-2 and checkpoint gene network in PCNSL. Nevertheless, we should conceive and develop innovative methods (e.g., cell-based cancer reprograming of cancer-cell themselves^43^) as an alternative to conventional immunotherapy with a checkpoint blockade.

## Methods

### Patients and materials

A total of 31 patients with PCNSL were enrolled. Patients were diagnosed according to the WHO classification^1,2^ and treated at Chiba University, Toyama Prefectural Central Hospital, Wakayama Medical University School of Medicine, and Yamaguchi University. This study was approved by the Ethics Committee of Kyoto Prefectural University of Medicine (RBMR-G-146) that covered recruitment of patients from other centers. Prior informed consents were obtained from all patients. Biopsy or resected tumor tissues immediately snap-frozen were collected. The experiments were performed in accordance with the institutional guidelines.

### NGS

Total RNAs were extracted from 100 mg of tumor biopsies or resected tissues using Isogen II (Nippongene). The quality of the extracted RNA was verified with the Bioanalyzer System using RNA Pico Chips (Agilent Technologies). NGS was performed using the Illumina HiSeq2000/2500 platform with a standard 124-bp paired-end read protocol^44,45^.

### Clustering analysis

Expression of genes of interests in the 31 PCNSL specimens was clustered with the hierarchical method using the JMP built-in modules (SAS Institute, Inc.)^22^.

### Kaplan-Meier survival analysis

The Kaplan-Meier analysis was performed to estimate survival distributions for subgroups with the log-rank test using the JMP built-in modules (SAS Institute Inc.)^22^.

### Random survival forest analysis

Random survival forest analysis was used to determine the factors with variable importance distinguishing the expression of transcript variants with NGS raw data^46,47^. Briefly, the values of variable importance reflected the relative contribution of each variable to the prediction for OS, and they were estimated by randomly permuting the values and recalculating the predictive accuracy of the model, which were expressed as the log rank test statistics. The method was implemented by using the randomForestSRC package of the statistical software R.

### Cox proportional hazards analysis

The association of expression of genes of interests with OS was evaluated by multivariable analyses with clinical characteristics as other predictors using the Cox proportional hazards regression model using the JMP built-in modules (SAS Institute Inc.)^46^.

### Multivariable correlation coefficient analysis

Correlation among variables were analyzed by the graphical lasso using the glasso package in R^48,49^. Correlations between pairs of variables were analyzed using the JMP built-in modules (SAS Institute, Inc.)^15^. Briefly, the correlations and multivariable analyses with multidimensional behavior of variables returned Pearson’s rank correlation coefficient values (r), in addition to the statistical significances with nonparametric analyses by the methods of Spearman, Kendall rank distance, and Hoeffding’s test of independence.

### GSEA

GSEA was performed using a dataset constructed by differential expression of genes defined by FPKM (FDR < 0.01)^43^. Differentially expressed genes were detected using the edgeR in Bioconductor package (http://bioconductor.org/packages/release/bioc/html/edgeR.html), followed by survey of pathways using the KEGG (https://www.genome.jp/kegg/).

### DLBCL dataset

A dataset of 47 patients with DLBCL available for OS and gene expression data (RNA-Seq) were collected from The Cancer Genome Atlas (TCGA) (https://tcga-data.nci.nih.gov/docs/publications/tcga/?) via the cBioPortal for Cancer Genomics (https://www.cbioportal.org/)^50^.

### Gene annotation

Genes of interests were annotated online at the GOstat (http://gostat.wehi.edu.au/) and the DAVID (https://david.ncifcrf.gov/)^50^.

### Statistics

Statistical analysis was performed using the JMP built-in modules (SAS Institute Inc.)^50^. *p*-value < 0.05 was considered statistically significant.

## Supplementary information

Supplementary Information

## Data Availability

The datasets generated during this study are available from the corresponding author on suitable request form.
