# Supplementary material for: Differential expression of individual transcript variants of PD-1 and PD-L2 genes on Th-1/Th-2 status is guaranteed for prognosis prediction in PCNSL
Source: Sci Rep. 2019 Jul 10;9:10004. doi: 10.1038/s41598-019-46473-5 (PMC6620277; doi:10.1038/s41598-019-46473-5)
Supplement: Supplementary file 1 — Supplementary Information [file 41598_2019_46473_MOESM1_ESM.pdf]

Differential expression of individual transcript variants of PD-1 and PD-L2 genes on Th-1/Th-2 status is guaranteed for prognosis prediction in PCNSL

Yasuo Takashima[1], Atsushi Kawaguchi[2], Ryuichi Sato[3,†], Kenichi Yoshida[4,†], Azusa Hayano[1], Jumpei Homma[5], Junya Fukai[6], Yasuo Iwadate[7], Koji Kajiwara[8], Shin Ishizawa[9], Hiroaki Hondoh[5], Masakazu Nakano[3], Seishi Ogawa[4], Kei Tashiro[3], and Ryuya Yamanaka[1,‡]

[1]Laboratory of Molecular Target Therapy for Cancer, Graduate School of Medical Science, Kyoto Prefectural University of Medicine, Kyoto, Japan
[2]Center for Comprehensive Community Medicine, Faculty of Medicine, Saga University, Saga, Japan
[3]Department of Genomic Medical Sciences, Graduate School of Medical Science, Kyoto Prefectural University of Medicine, Kyoto, Japan
[4]Department of Pathology and Tumor Biology, Graduate School of Medicine, Kyoto University, Kyoto, Japan
[5]Department of Neurosurgery, Toyama Prefectural Central Hospital, Toyama, Japan
[6]Department of Neurological Surgery, Wakayama Medical University School of Medicine, Wakayama, Japan
[7]Department of Neurosurgery, Graduate School of Medical Sciences, Chiba University, Chiba, Japan
[8]Department of Neurosurgery, Graduate School of Medical Sciences, Yamaguchi University, Ube, Yamaguchi, Japan
[9]Department of Pathology, Toyama Prefectural Central Hospital, Toyama, Japan

†These authors contributed equally to this work.
‡To whom correspondence should be addressed.

Corresponding author address:
Prof. Ryuya Yamanaka, Ph.D., M.D.
Laboratory of Molecular Target Therapy for Cancer, Graduate School of Medical Science, Kyoto Prefectural University of Medicine, 465 Kajii-cho, Kawaramachi-Hirokoji, Kamigyo-ku, Kyoto 602-8566, Japan.
E-mail; ryaman@koto.kpu-m.ac.jp.

Total number of supplementary materials: 21
Supplementary figure legends: 1
Supplementary references: 1
Supplementary figures: 14
Supplementary tables: 5

Supplementary Figure Legends

Suppl. Fig. S1. Kaplan-Meier survival analysis for primary central nervous system lymphoma (PCNSL) specimens. (**A**) PCNSL specimens (n = 31). (**B**) Gender. (**C**) Age by 60. (**D**) Karnofsky Performance status (KPS) by 70. (**E**) Memorial Sloan Kettering Cancer Center (MSKCC) score 1, 2, and 3. (**F**) International Extranodal Lymphoma Study Group (IELSG) 0-1, 2-3, and 4-5. (**G**) Chemotherapy including polychemotherapy, high dose-methotrexate (HD-MTX), and ionizing radiation (IR). Hazard ratio (HR) with 95% confidence interval (CI). OS; overall survival.

Suppl. Fig. S2. Expression of immune checkpoint genes in primary central nervous system lymphoma (PCNSL). Clustered expression of 84 transcript variants derived from 62 genes related to T helper cell type 1/2 (Th-1/Th-2) status and stimulatory and inhibitory checkpoints are shown in heat map. Hierarchical method was used. Relatively high and low expression are indicated by red and green, respectively.

Suppl. Fig. S3. Kaplan-Meier analysis on the expression levels of representative genes for stimulatory checkpoints. The patients were divided into two subgroups with high (black line) and low (red line) expression by the median expression of the transcript variants of IL2RB, TNFRSF18, CD27, CD40, and TNFRSF4. (**A**) Higher expression of the transcripts indicates poor prognoses. (**B**) Lower expression of the transcripts indicates poor prognoses. Hazard ratio (HR) with 95% confidence interval (CI) and *p*-value from log-rank test were calculated. OS; overall survival.

Suppl. Fig. S4. Kaplan-Meier analysis on the expression levels of representative genes for inhibitory checkpoints. The patients were divided into two subgroups with high (black line) and low (red line) expression by the median expression of the transcript variants of ADORA2A, BTLA, PDCD1LG2 (also known as CD273, PD-L2, or B7-DC), CD274 (also known as PDCD1LG1, PD-L1, or B7-H1), HAVCR2, IDO1. (**A**) Higher expression of the transcripts indicates poor prognoses. (**B**) Lower expression of the transcripts indicates poor prognoses. Hazard ratio (HR) with 95% confidence interval (CI) and *p*-value from log-rank test were calculated. OS; overall survival.

Suppl. Fig. S5. Comparative expression analysis of stimulatory checkpoint genes on the balance of T helper cell type 1/2 (Th-1/Th-2) status in primary central nervous system lymphoma (PCNSL). The box-whisker plots of the expression of stimulatory immune checkpoint genes. The PCNSL patients were divided into four subgroups, including Th-1^{high}Th-2^{high}, Th-1^{high}Th-2^{low}, Th-1^{low}Th-2^{high}, and Th-1^{low}Th-2^{low}.

Suppl. Fig. S6. Comparative expression analysis of inhibitory checkpoint genes on the balance of T helper cell type 1/2 (Th-1/Th-2) status in primary central nervous system lymphoma (PCNSL). The box-whisker plots of the expression of inhibitory immune checkpoint genes. The PCNSL patients were divided into four subgroups,

including Th-1^{high}Th-2^{high}, Th-1^{high}Th-2^{low}, Th-1^{low}Th-2^{high}, and Th-1^{low}Th-2^{low}.

Suppl. Fig. S7. Kaplan-Meier analysis for higher expression levels of representative genes for inhibitory checkpoints. The patients were divided into two subgroups with high (black line) and low (red line) expression by the cutoff score of the expression of the transcript variants, including CD160, CD276, CD86, CD96, CEACAM1, CTLA4, HAVCR2, LGALS3, PDCD1LG2, PVR, TIGIT, TMIGD2, TNFRSF14, KIR3DL1, and VTCN1. Higher expression of the transcript variants indicates poor prognoses. Hazard ratio (HR) with 95% confidence interval (CI) and *p*-value from log-rank test were calculated. OS, overall survival.

Suppl. Fig. S8. Kaplan-Meier analysis for lower expression levels of representative genes for inhibitory checkpoints. The patients were divided into two subgroups with high (black line) and low (red line) expression by the cutoff score of the expression of the transcript variants, including BTLA, C10orf54, CD160, CD274, CD80, CD86, CD96, CEACAM1, IDO1, LGALS9, PVR, KIR3DL1-003, and VTCN1-001/002. Lower expression of the transcript variants indicates poor prognoses. Hazard ratio (HR) with 95% confidence interval (CI) and *p*-value from log-rank test were calculated. OS, overall survival.

Suppl. Fig. S9. Correlation analysis among gene expression for T helper cell type 1/2 (Th-1/Th-2) status and immune checkpoint in primary central nervous system lymphoma (PCNSL). (**A**) Statistical analyses were performed with the methods by Pearson's correlation coefficient (r), in addition to nonparametric analyses by the methods of Hoeffding, Kendall, and Spearman. Correlation coefficient values with $p < 0.05$ are shown. Right and bottom numbers indicate the numbers of correlation between variable 1 (column; n = 38) and variable 2 (row; n = 35). (**B**) Non-parametric analyses by the methods of Spearman, Hoeffding, and Kendall in the representative genes. High and low correlations in each analysis are indicated in red and green, respectively.

Suppl. Fig. S10. Differential expression of genes related to T cell receptor signaling pathway (KEGG ID: hsa04660) detected in PCNSL.[97-99]

Suppl. Fig. S11. Differential expression of genes related to cytokine-cytokine receptor interaction (KEGG ID: hsa04060) detected in PCNSL.[97-99]

Suppl. Fig. S12. Differential expression of genes related to cell adhesion molecules (KEGG ID: hsa04514) detected in PCNSL.[97-99]

Suppl. Fig. S13. Helper T cell balance and expression of immune checkpoint genes in DLBCL. (**A-D**) Survival distribution of the subgroups divided by the scores of Th-1 (**A**), Th-2 (**B**), and stimulatory (**C**) and inhibitory checkpoint genes (**D**), respectively. (**E**) Th-1/Th-2 balance in DLBCL data set. R^2, correlation coefficient. (**F-G**)

(**F**)Survival distribution of the subgroups divided by the Th-1/Th-2 balance. (**G**) Hazard ratio (HR) with 95% confidence interval (CI) compared with the $Th1^{Low}Th2^{High}$ subgroup. (**H-I**) Co-expression of immune checkpoint genes on Th balances in DLBCL data set. (**H**) Stimulatory checkpoint genes. (**I**) Inhibitory checkpoint genes. OS; overall survival, R^2; correlation coefficient, n.s.; not significant. Subgroups were divided by the median scores.

Suppl. Fig. S14. Survival distribution of the subgroups of DLBCL patients. (**A**) Overall survival (OS) of 47 DLBCL patients. (**B-I**) Survival distributions of the DLBCL subgroups divided by the median expression of CD40 (**B**), CD70 (**C**), CD80 (**D**), CEACAM1 (**E**), LGALS9 (**F**), LAG3 (**G**), PDCD1 (**H**), and PDCD1LG2 (**I**). Hazard ratios (HR) with 95% CI are shown. OS, overall survival.

Supplementary References

51. Bielinska AU, Makidon PE, Janczak KW, Blanco LP, Swanson B, Smith DM, Pham T, Szabo Z, Kukowska-Latallo JF, Baker JR. Distinct pathways of humoral and cellular immunity induced with the mucosal administration of a nanoemulsion adjuvant. J Immunol. 2014; 192(6):2722-33.
52. Bogunia-Kubik K, Polak M, Lange A. TNF polymorphisms are associated with toxic but not with aGVHD complications in the recipients of allogeneic sibling haematopoietic stem cell transplantation. Bone Marrow Transplant. 2003 Sep;32(6):617-22.
53. Cervera-Carrascon V, Siurala M, Santos JM, Havunen R, Tähtinen S, Karell P, Sorsa S, Kanerva A, Hemminki A. TNFa and IL-2 armed adenoviruses enable complete responses by anti-PD-1 checkpoint blockade. Oncoimmunology. 2018 Apr 9;7(5):e1412902.
54. Choi IK, Wang Z, Ke Q, Hong M, Qian Y, Zhao X, Liu Y, Kim HJ, Ritz J, Cantor H, Rajewsky K, Wucherpfennig KW, Zhang B. Signaling by the Epstein-Barr virus LMP1 protein induces potent cytotoxic CD4+ and CD8+ T cell responses. Proc Natl Acad Sci U S A. 2018 Jan 23;115(4):E686-E695.
55. Chou FC, Chen HY, Kuo CC, Sytwu HK. Role of Galectins in Tumors and in Clinical Immunotherapy. Int J Mol Sci. 2018 Feb 1;19(2). pii: E430.
56. Dankner M, Gray-Owen SD, Huang YH, Blumberg RS, Beauchemin N. CEACAM1 as a multi-purpose target for cancer immunotherapy. Oncoimmunology. 2017 May 16;6(7):e1328336.
57. Dawany N, Parzych EM, Showe LC, Ertl HC. Age-related changes in the gene expression profile of antigen-specific mouse CD8+ T cells can be partially reversed by blockade of the BTLA/CD160 pathways during vaccination. Aging (Albany NY). 2016 Nov 9;8(12):3272-3297.
58. Di Meo S, Airoldi I, Sorrentino C, Zorzoli A, Esposito S, Di Carlo E. Interleukin-30 expression in prostate cancer and its draining lymph nodes correlates with advanced grade and stage. Clin Cancer Res. 2013; 20(3):585-94.
59. Dougall WC, Kurtulus S, Smyth MJ, Anderson AC. TIGIT and CD96: new checkpoint receptor targets for cancer immunotherapy. Immunol Rev. 2017 Mar;276(1):112-120.
60. Fourcade J, Sun Z, Chauvin JM, Ka M, Davar D, Pagliano O, Wang H, Saada S, Menna C, Amin R, Sander C, Kirkwood JM, Korman AJ, Zarour HM. CD226 opposes TIGIT to disrupt Tregs in melanoma. JCI Insight. 2018 Jul 26;3(14). pii: 121157.
61. Friedlander P, Wood K, Wassmann K, Christenfeld AM, Bhardwaj N5, Oh WK. A whole-blood RNA transcript-based gene signature is associated with the development of CTLA-4 blockade-related diarrhea in patients with advanced melanoma treated with the checkpoint inhibitor tremelimumab. J Immunother Cancer. 2018 Sep 18;6(1):90.
62. Ghosh D, Curtis AD 2nd, Wilkinson DS, Mannie MD. Depletion of CD4+ CD25+ regulatory T cells confers susceptibility to experimental autoimmune encephalomyelitis (EAE) in GM-CSF-deficient Csf2-/- mice. J Leukoc Biol. 2016 Oct;100(4):747-760.

63. Gieseke F, Kruchen A, Tzaribachev N, Bentzien F, Dominici M, Müller I. Proinflammatory stimuli induce galectin-9 in human mesenchymal stromal cells to suppress T-cell proliferation. Eur J Immunol. 2013 Oct;43(10):2741-9.
64. Gordon-Alonso M, Hirsch T, Wildmann C, van der Bruggen P. Galectin-3 captures interferon-gamma in the tumor matrix reducing chemokine gradient production and T-cell tumor infiltration. Nat Commun. 2017 Oct 6;8(1):793.
65. Goulding J, Abboud G, Tahiliani V, Desai P, Hutchinson TE, Salek-Ardakani S. CD8 T cells use IFN-γ to protect against the lethal effects of a respiratory poxvirus infection. J Immunol. 2014; 192(11):5415-25.
66. Gubin MM, Zhang X, Schuster H, Caron E, Ward JP, Noguchi T, Ivanova Y, Hundal J, Arthur CD, Krebber WJ, Mulder GE, Toebes M, Vesely MD, Lam SS, Korman AJ, Allison JP, Freeman GJ, Sharpe AH, Pearce EL, Schumacher TN, Aebersold R, Rammensee HG, Melief CJ, Mardis ER, Gillanders WE, Artyomov MN, Schreiber RD. Checkpoint blockade cancer immunotherapy targets tumour-specific mutant antigens. Nature. 2014 Nov 27;515(7528):577-81.
67. Halim TYF, Rana BMJ, Walker JA, Kerscher B, Knolle MD, Jolin HE, Serrao EM, Haim-Vilmovsky L, Teichmann SA, Rodewald HR, Botto M, Vyse TJ, Fallon PG, Li Z, Withers DR, McKenzie ANJ. Tissue-Restricted Adaptive Type 2 Immunity Is Orchestrated by Expression of the Costimulatory Molecule OX40L on Group 2 Innate Lymphoid Cells. Immunity. 2018 Jun 19;48(6):1195-1207.e6.
68. Harjunpää H, Blake SJ, Ahern E, Allen S, Liu J, Yan J, Lutzky V, Takeda K, Aguilera AR, Guillerey C, Mittal D, Li XY, Dougall WC, Smyth MJ, Teng MWL. Deficiency of host CD96 and PD-1 or TIGIT enhances tumor immunity without significantly compromising immune homeostasis. Oncoimmunology. 2018 Mar 26;7(7):e1445949.
69. Hayano A, Komohara Y, Takashima Y, Takeya H, Homma J, Fukai J, Iwadate Y, Kajiwara K, Ishizawa S, Hondoh H, Yamanaka R. Programmed Cell Death Ligand 1 Expression in Primary Central Nervous System Lymphomas: A Clinicopathological Study. Anticancer Res. 2017 Oct;37(10):5655-5666.
70. Heuer JG, Tucker-McClung C, Hock RA. Neuroblastoma cells expressing mature IL-18, but not proIL-18, induce a strong and immediate antitumor immune response. J Immunother. 1999 Jul;22(4):324-35.
71. Hoelzinger DB, Dominguez AL, Cohen PA, Gendler SJ. Inhibition of adaptive immunity by IL9 can be disrupted to achieve rapid T-cell sensitization and rejection of progressive tumor challenges. Cancer Res. 2014 Dec 1;74(23):6845-55.
72. Huang YH, Zhu C, Kondo Y, Anderson AC, Gandhi A, Russell A, Dougan SK, Petersen BS, Melum E, Pertel T, Clayton KL, Raab M, Chen Q, Beauchemin N, Yazaki PJ, Pyzik M, Ostrowski MA, Glickman JN, Rudd CE, Ploegh HL, Franke A, Petsko GA, Kuchroo VK, Blumberg RS. CEACAM1 regulates TIM-3-mediated tolerance and exhaustion. Nature. 2015 Jan 15;517(7534):386-90.
73. Janakiram M, Chinai JM, Zhao A, Sparano JA, Zang X. HHLA2 and TMIGD2: new immunotherapeutic targets of the B7 and CD28 families. Oncoimmunology. 2015 Apr 14;4(8):e1026534.
74. Jounaidi Y, Cotten JF, Miller KW, Forman SA. Tethering IL2 to Its Receptor IL2Rβ Enhances Antitumor

Activity and Expansion of Natural Killer NK92 Cells. Cancer Res. 2017 Nov 1;77(21):5938-5951.

75. Kim YH, Shin SM, Choi BK, Oh HS, Kim CH, Lee SJ, Kim KH, Lee DG, Park SH, Kwon BS. Authentic GITR Signaling Fails To Induce Tumor Regression unless Foxp3+ Regulatory T Cells Are Depleted. J Immunol. 2015 Nov 15;195(10):4721-9.
76. Lee YH, Martin-Orozco N, Zheng P, Li J, Zhang P, Tan H, Park HJ, Jeong M, Chang SH, Kim BS, Xiong W, Zang W, Guo L, Liu Y, Dong ZJ, Overwijk WW, Hwu P, Yi Q, Kwak L, Yang Z, Mak TW, Li W, Radvanyi LG, Ni L, Liu D, Dong C. Inhibition of the B7-H3 immune checkpoint limits tumor growth by enhancing cytotoxic lymphocyte function. Cell Res. 2017 Aug;27(8):1034-1045.
77. Li J, Lee Y, Li Y, Jiang Y, Lu H, Zang W, Zhao X, Liu L, Chen Y, Tan H, Yang Z, Zhang MQ, Mak TW, Ni L, Dong C. Co-inhibitory Molecule B7 Superfamily Member 1 Expressed by Tumor-Infiltrating Myeloid Cells Induces Dysfunction of Anti-tumor CD8+ T Cells. Immunity. 2018 Apr 17;48(4):773-786.e5.
78. Ly CL, Nores GDG, Kataru RP, Mehrara BJ. T helper 2 differentiation is necessary for development of lymphedema. Transl Res. 2018 Dec 21. pii: S1931-5244(18)30234-2.
79. McCracken MK, Christofferson RC, Chisenhall DM, Mores CN. Analysis of early dengue virus infection in mice as modulated by Aedes aegypti probing. J Virol. 2013; 88(4):1881-9.
80. Melssen M, Slingluff CL Jr. Vaccines targeting helper T cells for cancer immunotherapy. Curr Opin Immunol. 2017 Aug;47:85-92.
81. Mitzel DN, Jaramillo RJ, Stout-Delgado H, Senft AP, Harrod KS. Human metapneumovirus inhibits the IL-6-induced JAK/STAT3 signalling cascade in airway epithelium. J Gen Virol. 2013; 95(Pt 1):26-37.
82. Ochiai S, Jagot F, Kyle RL, Hyde E, White RF, Prout M, Schmidt AJ, Yamane H, Lamiable O, Le Gros G, Ronchese F. Thymic stromal lymphopoietin drives the development of IL-13+ Th2 cells. Proc Natl Acad Sci U S A. 2018 Jan 30;115(5):1033-1038.
83. Ock SA, Lee J, Oh KB, Hwang S, Yun IJ, Ahn C, Chee HK, Kim H, Park JB, Kim SJ, Kim Y, Im GS, Park E. Molecular immunology profiles of monkeys following xenografting with the islets and heart of α-1,3-galactosyltransferase knockout pigs. Xenotransplantation. 2016 Sep;23(5):357-69.
84. Rotte A, Jin JY, Lemaire V. Mechanistic overview of immune checkpoints to support the rational design of their combinations in cancer immunotherapy. Ann Oncol. 2018 Jan 1;29(1):71-83.
85. Schmitt N, Ueno H. Regulation of human helper T cell subset differentiation by cytokines. Curr Opin Immunol. 2015 Jun;34:130-6.
86. Shen YL, Gan Y, Gao HF, Fan YC, Wang Q, Yuan H, Song YF, Wang JD, Tu H. TNFSF9 exerts an inhibitory effect on hepatocellular carcinoma. J Dig Dis. 2017 Jul;18(7):395-403.
87. Sideras K, Biermann K, Yap K, Mancham S, Boor PPC, Hansen BE, Stoop HJA, Peppelenbosch MP, van Eijck CH, Sleijfer S, Kwekkeboom J, Bruno MJ. Tumor cell expression of immune inhibitory molecules and tumor-infiltrating lymphocyte count predict cancer-specific survival in pancreatic and ampullary cancer. Int J Cancer. 2017 Aug 1;141(3):572-582.
88. Sim GC, Radvanyi L. The IL-2 cytokine family in cancer immunotherapy. Cytokine Growth Factor Rev. 2014

Aug;25(4):377-90.

89. Stamm H, Wellbrock J, Fiedler W. Interaction of PVR/PVRL2 with TIGIT/DNAM-1 as a novel immune checkpoint axis and therapeutic target in cancer. Mamm Genome. 2018 Dec;29(11-12):694-702.

90. Takashima Y, Kawaguchi A, Kanayama T, Hayano A, Yamanaka R. Correlation between lower balance of Th2 helper T-cells and expression of PD-L1/PD-1 axis genes enables prognostic prediction in patients with glioblastoma. Oncotarget. 2018 Apr 10;9(27):19065-19078.

91. Tanoue T, Morita S, Plichta DR, Skelly AN, Suda W, Sugiura Y, Narushima S, Vlamakis H, Motoo I, Sugita K, Shiota A, Takeshita K, Yasuma-Mitobe K, Riethmacher D, Kaisho T, Norman JM, Mucida D, Suematsu M, Yaguchi T, Bucci V, Inoue T, Kawakami Y, Olle B, Roberts B, Hattori M, Xavier RJ, Atarashi K, Honda K. A defined commensal consortium elicits CD8 T cells and anti-cancer immunity. Nature. 2019 Jan;565(7741):600-605.

92. Till SJ, Raynsford EJ, Reynolds CJ, Quigley KJ, Grzybowska-Kowalczyk A, Saggar LR, Goldstone A, Maillere B, Kwok WW, Altmann DM, Durham SR, Boyton RJ. Peptide-induced immune regulation by a promiscuous and immunodominant CD4T-cell epitope of Timothy grass pollen: a role of Cbl-b and Itch in regulation. Thorax. 2013; 69(4):335-45.

93. Xie S, Huang J, Qiao Q, Zang W, Hong S, Tan H, Dong C, Yang Z, Ni L. Expression of the inhibitory B7 family molecule VISTA in human colorectal carcinoma tumors. Cancer Immunol Immunother. 2018 Nov;67(11):1685-1694.

94. Yang Y. Cancer immunotherapy: harnessing the immune system to battle cancer. J Clin Invest. 2015 Sep;125(9):3335-7.

95. Zhao L, Zhu H, Han B, Wang L, Sun Y, Lu X, Huang C, Tan B, Chen C, Qin L. Influence of genetic polymorphisms of IL23R, STAT3, IL12B, and STAT4 on the risk of aplastic anemia and the effect of immunosuppressive therapy. Ann Hematol. 2018 Apr;97(4):685-695.

96. Zhu Y, Knolhoff BL, Meyer MA, Nywening TM, West BL, Luo J, Wang-Gillam A, Goedegebuure SP, Linehan DC, DeNardo DG. CSF1/CSF1R blockade reprograms tumor-infiltrating macrophages and improves response to T-cell checkpoint immunotherapy in pancreatic cancer models. Cancer Res. 2014 Sep 15;74(18):5057-69.

97. Kanehisa M, Sato Y, Furumichi M, Morishima K, Tanabe M. New approach for understanding genome variations in KEGG. Nucleic Acids Res. 2019 Jan 8;47(D1):D590-D595.

98. Kanehisa M, Furumichi M, Tanabe M, Sato Y, Morishima K. KEGG: new perspectives on genomes, pathways, diseases and drugs. Nucleic Acids Res. 2017 Jan 4;45(D1):D353-D361.

99. Kanehisa M, Goto S. KEGG: kyoto encyclopedia of genes and genomes. Nucleic Acids Res. 2000 Jan 1;28(1):27-30.

Suppl. Fig. S1

Takashima et al.

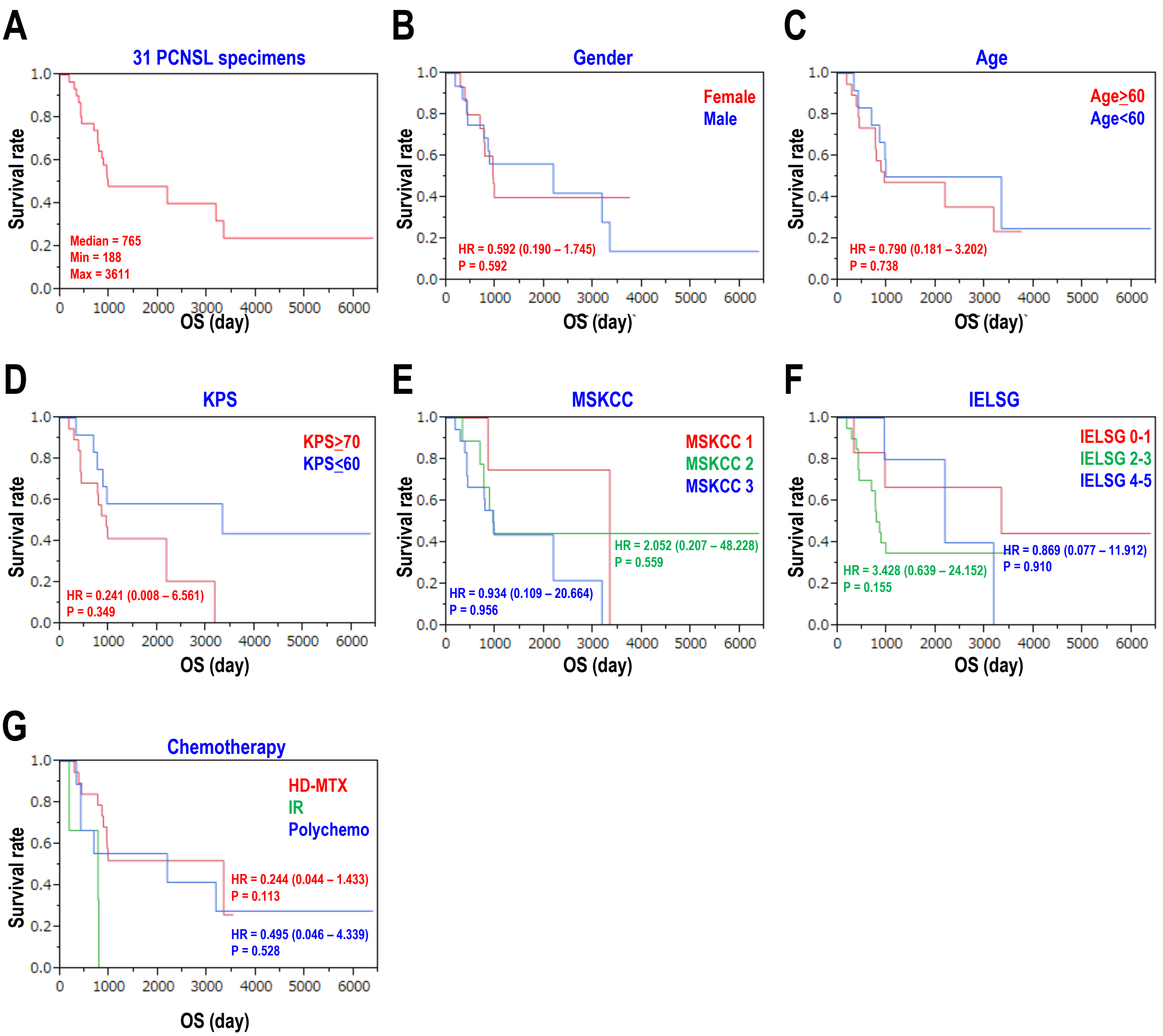

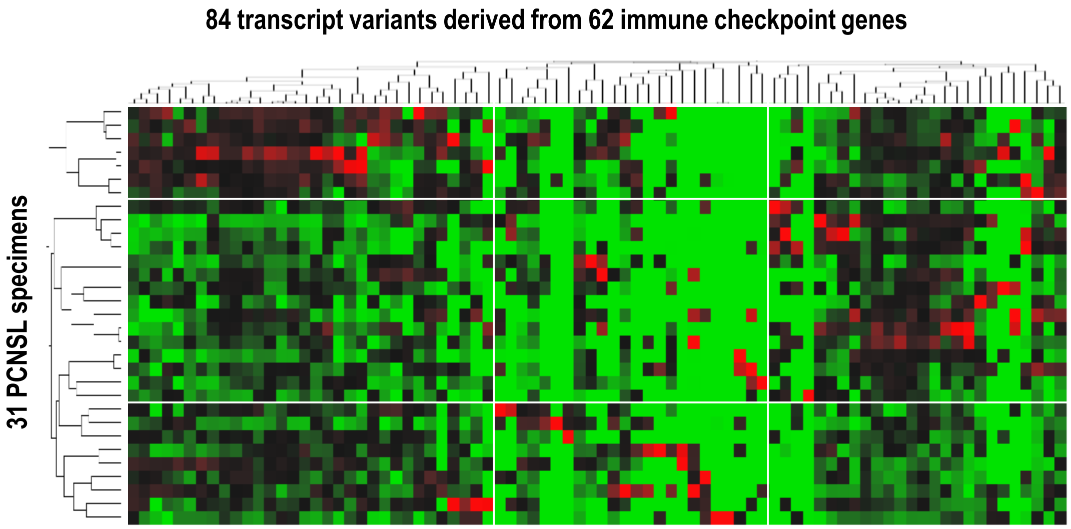
84 transcript variants derived from 62 immune checkpoint genes
31 PCNSL specimens

Suppl. Fig. S3

Takashima et al.

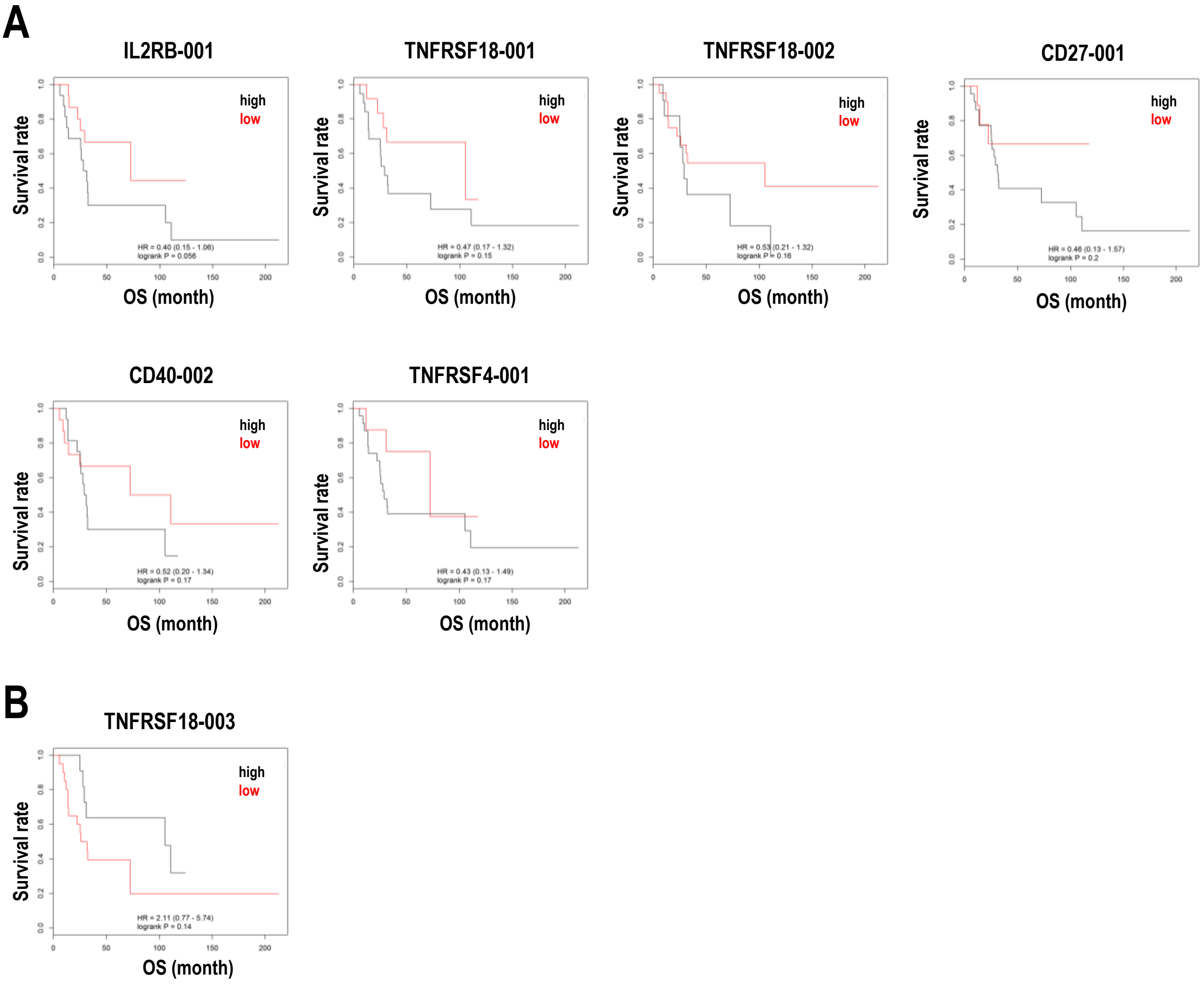

A

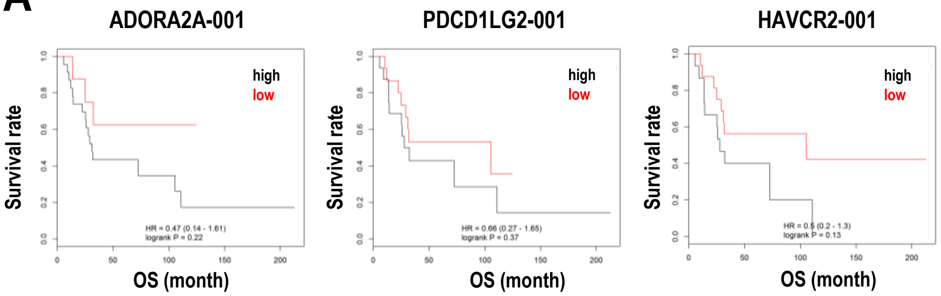
ADORA2A-001
high
low
HR = 0.47 (0.14 - 1.61)
logrank P = 0.22
PDCD1LG2-001
high
low
HR = 0.66 (0.27 - 1.65)
logrank P = 0.37
HAVCR2-001
high
low
HR = 0.5 (0.2 - 1.3)
logrank P = 0.13
Survival rate
OS (month)

B

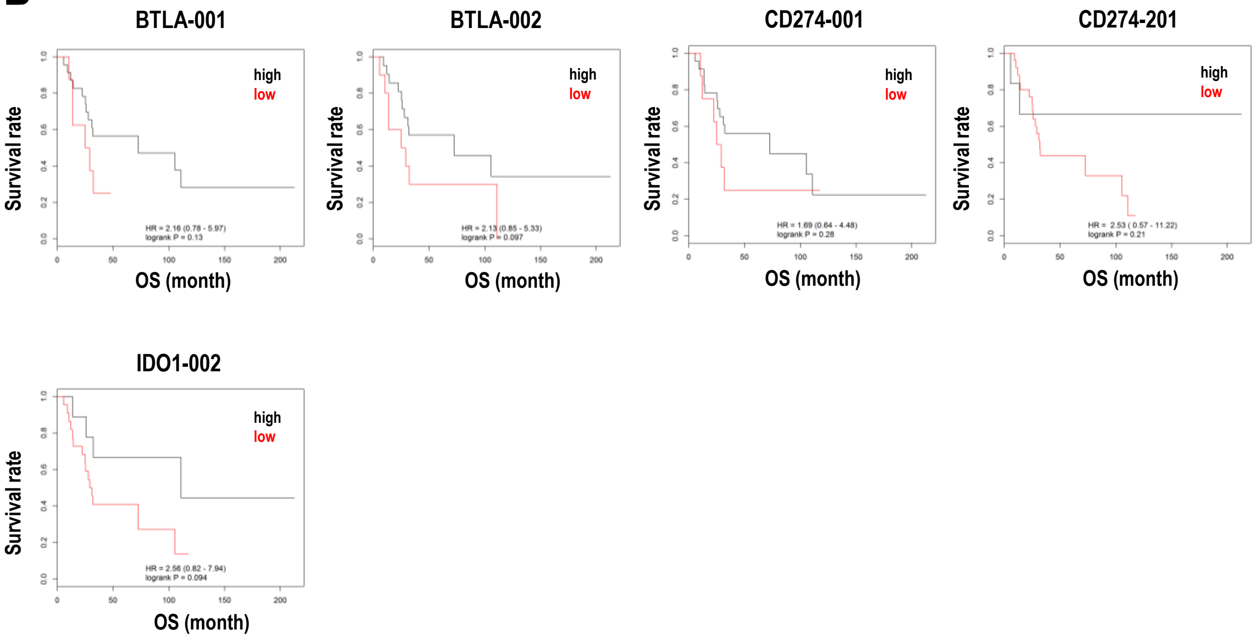
BTLA-001
high
low
HR = 2.16 (0.78 - 5.97)
logrank P = 0.13
BTLA-002
high
low
HR = 2.13 (0.85 - 5.33)
logrank P = 0.097
CD274-001
high
low
HR = 1.69 (0.64 - 4.48)
logrank P = 0.28
CD274-201
high
low
HR = 2.53 (0.57 - 11.22)
logrank P = 0.21
IDO1-002
high
low
HR = 2.56 (0.82 - 7.94)
logrank P = 0.094
Survival rate
OS (month)

Suppl. Fig. S5

Takashima et al.

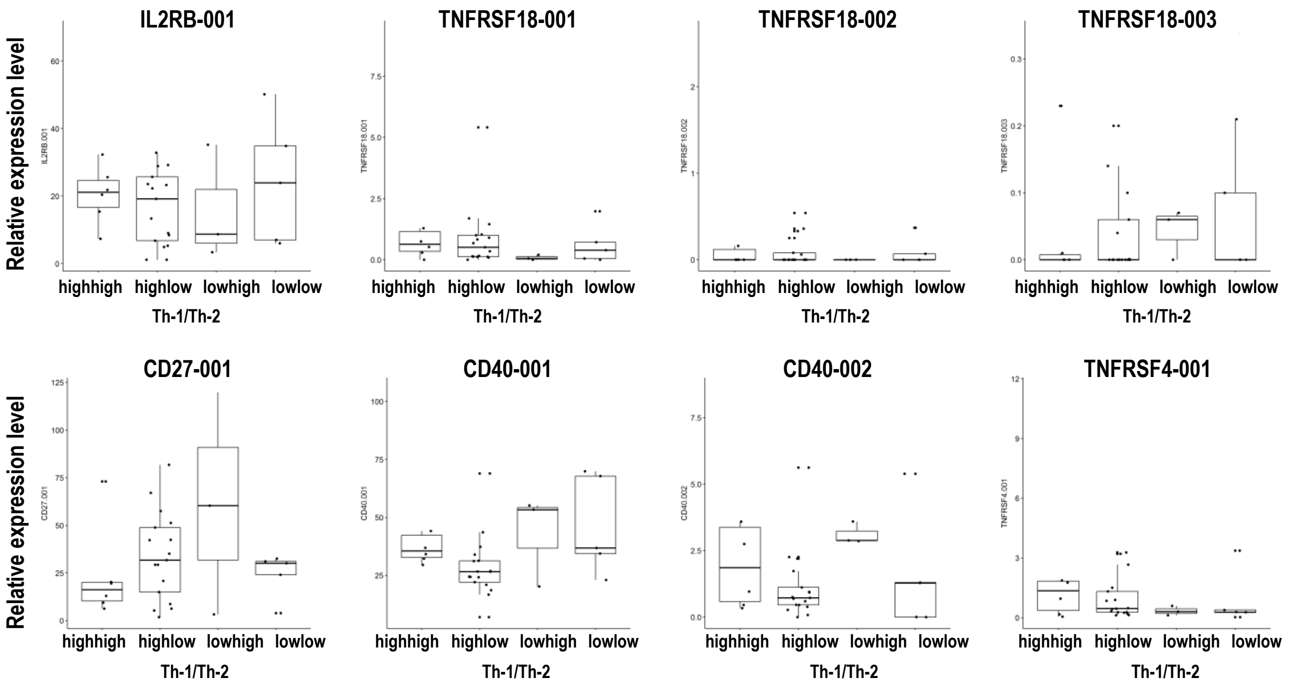

Suppl. Fig. S6 **Takashima et al.**

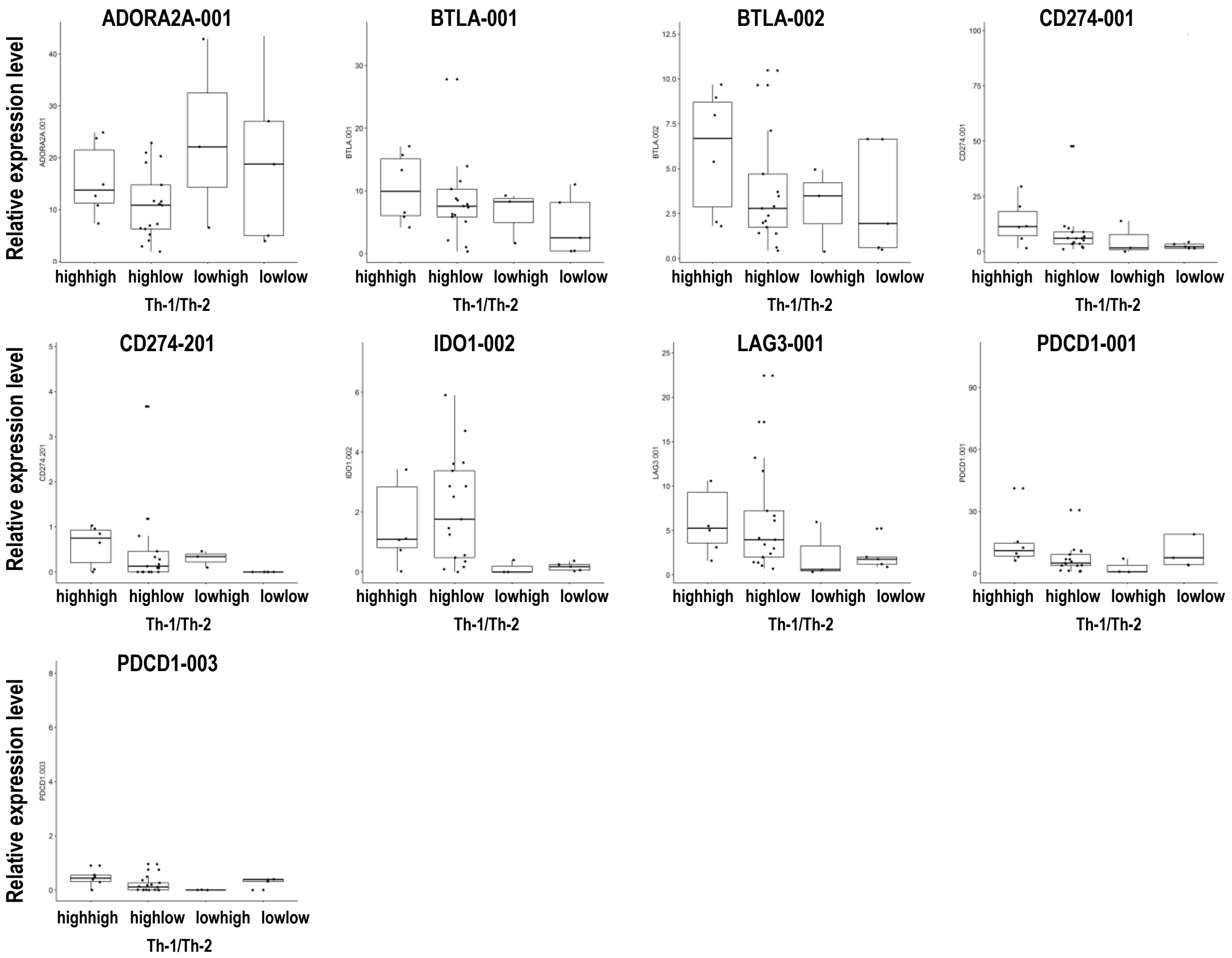

Suppl. Fig. S7 **Takashima et al.**

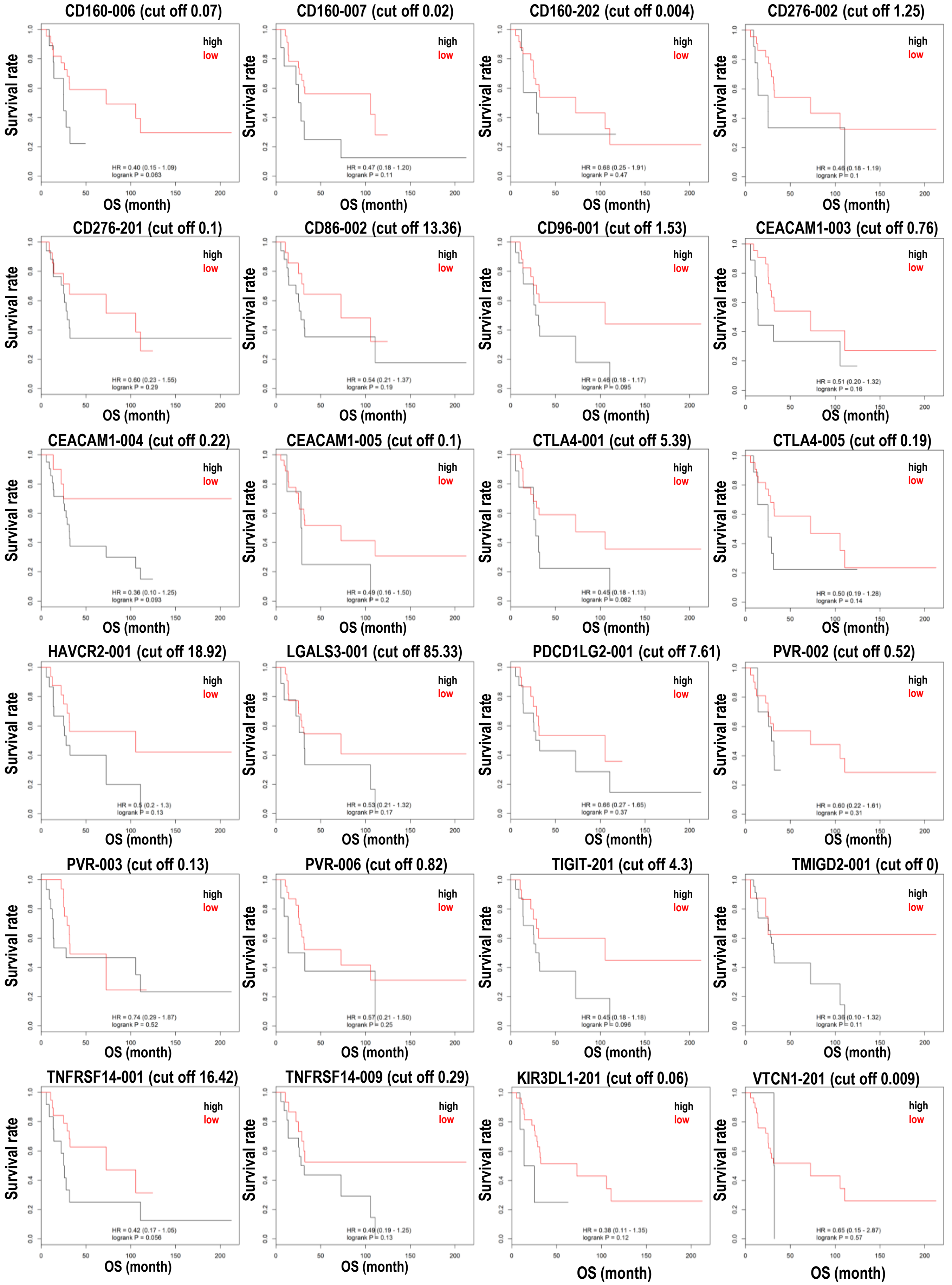

Suppl. Fig. S8 **Takashima et al.**

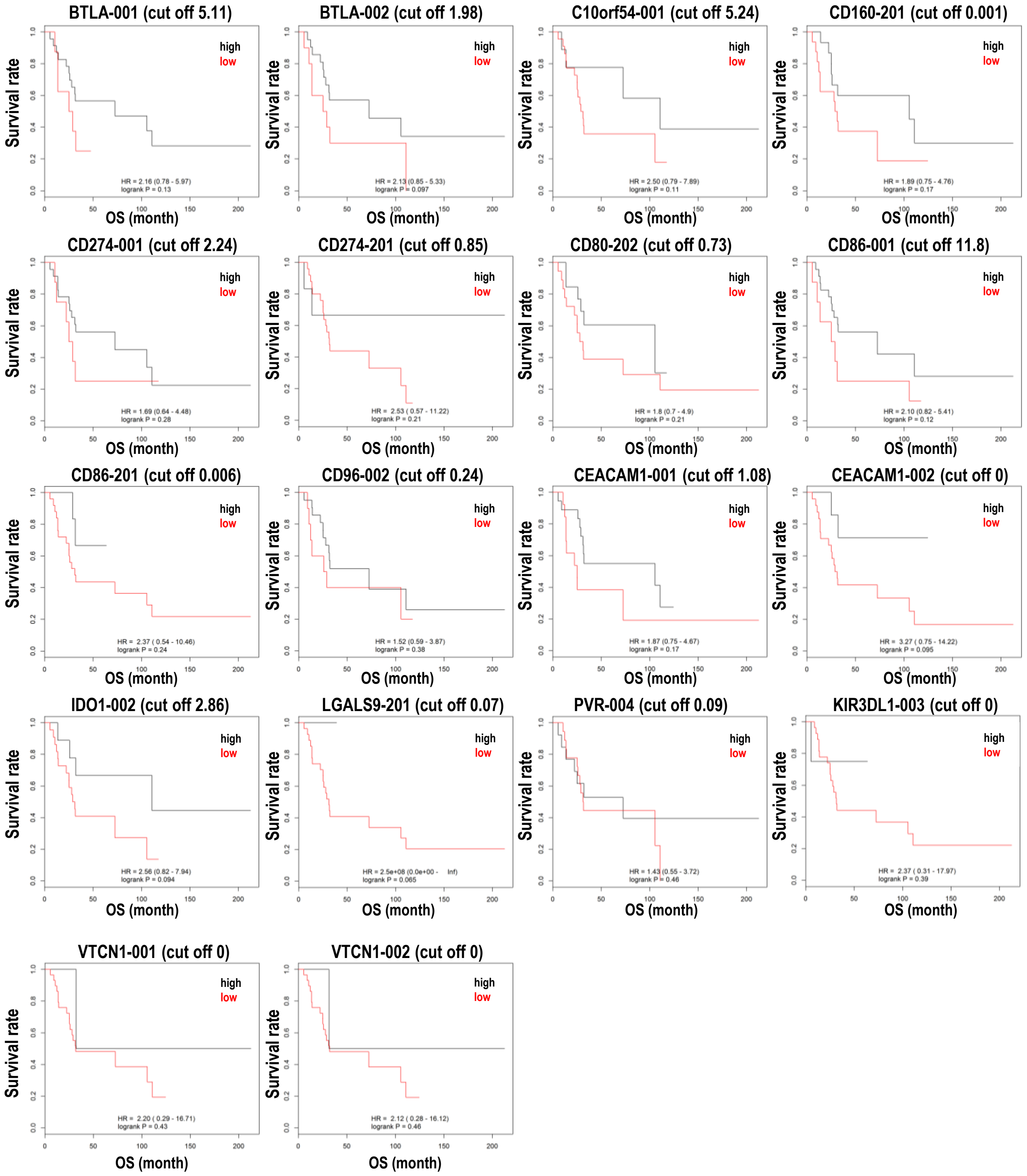

Suppl. Fig. S9

Takashima et al.

A

	CD28-001	STAT4-002	IFNG-001	CD4-001	CD28-202	TBX21-001	TNFRSF1A-001	STAT1-001	ICOS-001	GATA3-001	PDCD1LG2-001	STAT1-003	TNFRSF18-001	PDCD1LG2-201	TGFB3-001	CD40-001	STAT1-010	CTLA4-001	TNFSF4-002	TNFRSF1A-004	GATA3-002	HAVCR2-001	STAT1-002	STAT4-007	STAT1-011	CD40LG-001	PDCD1-001	TNFRSF4-001	TNFRSF9-001	TNFSF4-001	CD27-001	STAT6-001	BTLA-001	IL18R1-001	IL6-001	
HAVCR2-001	0.73	0.82	0.66	0.78	0.64	0.61	0.84	0.61	0.73	0.89	0.75	0.55		0.54	0.48	-0.51		0.74	0.72	0.46																18
LAG3-001	0.57	0.68	[illegible]	0.52	0.62	0.79	0.55		0.59	0.89			0.80					0.63			0.65	[illegible]						0.69								14
PDCD1LG2-001	0.58	0.63	0.70	0.82		0.47	0.73	0.70	0.56			0.64											0.55						0.60	0.48						12
ICOS-001	0.87	0.75	0.58	0.55	0.72	0.61	0.61	0.55		0.60											0.61															10
IDO1-002	0.47	0.59	0.78					0.75	0.52		0.63			0.59					0.59			0.46														9
CTLA4-001	0.78	0.71	0.55	0.62			0.60		0.89		0.60																				0.48					8
GATA3-002		0.62	0.47		0.70	0.65	0.55			0.66			0.54																							7
PDCD1LG2-201	0.55	0.53	0.56	0.53				0.73	0.49		0.73																									7
TNFRSF1A-001	0.69	0.74	0.50	0.90	0.62	0.61						0.67																								7
TBX21-001	0.62	0.69	0.59	0.49	0.69																			0.49												6
TNFRSF1A-004		0.49			0.57		0.52										0.46							0.64	0.47											6
GATA3-001	0.61	0.62			0.62	0.70							0.88																							5
STAT4-002	0.85		0.79	0.64	0.71			0.60																												5
TNFSF4-002	0.52	0.67	0.62					0.62	0.58																											5
CD276-002				0.63													0.48								0.74											3
STAT1-011				0.53								0.83					0.61																			3
STAT4-007	0.53	0.47	0.50																																	3
TGFB3-001							[illegible]													[illegible]												-0.58				3
TNFRSF18-001		0.48				0.58																				0.75										3
TNFSF4-001						0.50				0.75			0.92																							3
BTLA-002															-0.46																		0.84			2
CD4-001	0.63				0.50																															2
IFNG-001	0.61				0.66																															2
STAT1-001	0.52		0.71																																	2
STAT1-002			0.52					0.69																												2
STAT1-003				0.73																			[illegible]													2
ADORA2A-001																0.64																				1
CD274-001														0.55																						1
CD276-201															0.46																					1
CD28-202	0.70																																			1
CD40-002																0.78																				1
IL12RB2-001	0.59																																			1
IL18R1-201																																		0.67		1
IL6-201																																			0.47	1
PDCD1-002																											0.84									1
PDCD1-003																											0.95									1
STAT1-010																										0.50										1
TNFRSF4-001																																				0
	18	15	15	12	11	9	8	8	7	5	4	4	4	3	3	3	3	2	2	2	2	2	2	2	2	2	2	1	1	1	1	1	1	1	1	

B

Variable 2	CD28-001	BTLA-001	CD4-001	TNFRSF1A-001	IFNG-001	CD4-001	STAT1-001	CD28-001	PDCD1LG2-001	TBX21-001	CD28-001	PDCD1LG2-001	TNFRSF18-001	IFNG-001	STAT1-002	IFNG-001	IFNG-001	CD4-001	STAT1-002	CD4-001	IFNG-001	IL18R1-001	PDCD1LG2-001	STAT4-002	STAT4-002	IFNG-001	STAT1-003	IFNG-001	STAT4-002	STAT1-001				
Variable 1	ICOS-001	BTLA-002	TNFRSF1A-001	HAVCR2-001	STAT4-002	PDCD1LG2-001	PDCD1LG2-201	STAT4-002	HAVCR2-001	LAG3-001	TNFRSF18-001	CD274-001	ICOS-001	TBX21-001	STAT1-003	STAT1-002	PDCD1LG2-001	HAVCR2-001	STAT4-002	STAT1-003	IDO1-002	IL18R1-202	PDCD1LG2-201	ICOS-001	HAVCR2-001	PDCD1LG2-201	HAVCR2-001	STAT1-001	STAT4-007	IDO1-002	ave	med	min	max
Spearman's ρ	0.89	0.86	0.86	0.84	0.82	0.85	0.79	0.83	0.78	0.81	0.79	0.70	0.77	0.76	0.78	0.76	0.76	0.78	0.75	0.77	0.75	[illegible]	0.74	0.73	0.75	0.72	0.72	0.74	0.75	0.73	0.78	0.77	0.70	0.89
Hoeffding's D	0.39	0.39	0.38	0.32	0.33	0.30	0.34	0.30	0.33	0.30	0.27	0.34	[illegible]	[illegible]	0.22	0.23	0.22	0.20	[illegible]	0.21	0.21	0.19	0.21	0.22	0.21	0.25	0.23	0.21	0.20	0.22	0.26	0.24	0.19	0.39
Kendall's τ	0.70	0.71	0.70	0.69	0.66	0.64	0.63	0.63	0.63	0.64	0.58	0.59	0.59	0.59	0.57	0.57	0.57	[illegible]	0.56	0.57	0.56	0.56	0.56	0.57	0.55	0.53	0.52	0.51	0.54	0.54	0.59	0.57	0.51	0.71

Suppl. Fig. S10

Takashima et al.

hsa04660: T cell receptor signaling pathway

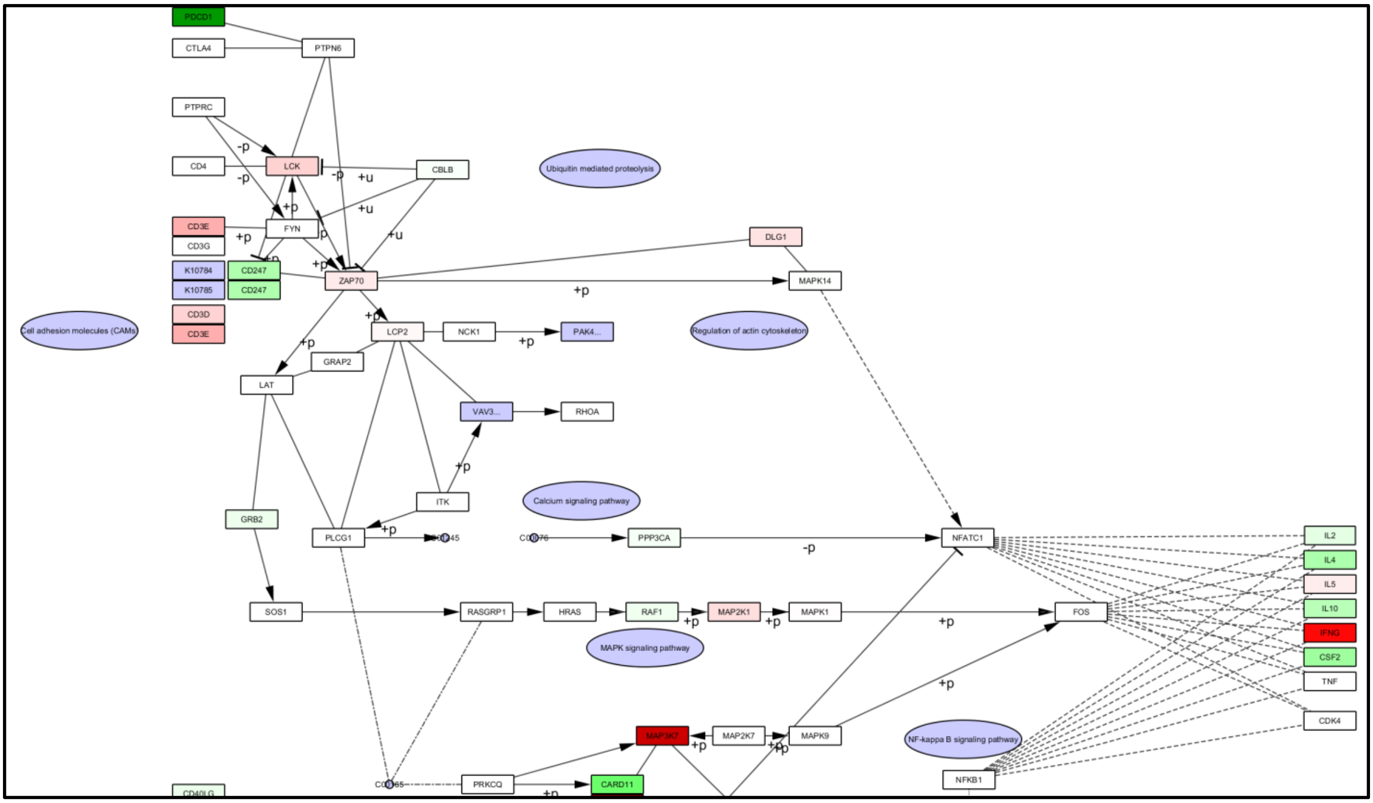

Suppl. Fig. S11

Takashima et al.

hsa04060: Cytokine-cytokine receptor interaction

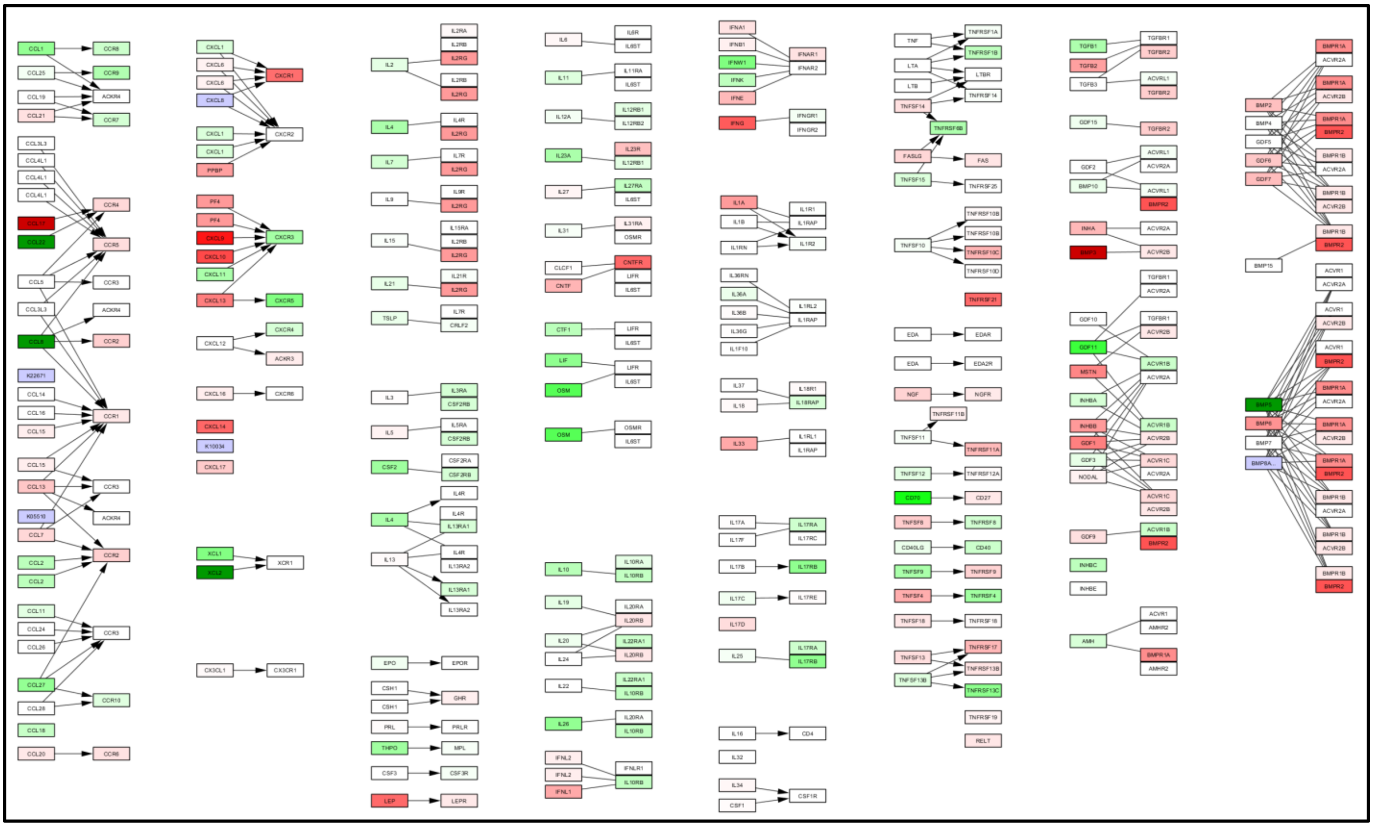

Suppl. Fig. S12

Takashima et al.

hsa04514: Cell adhesion molecules

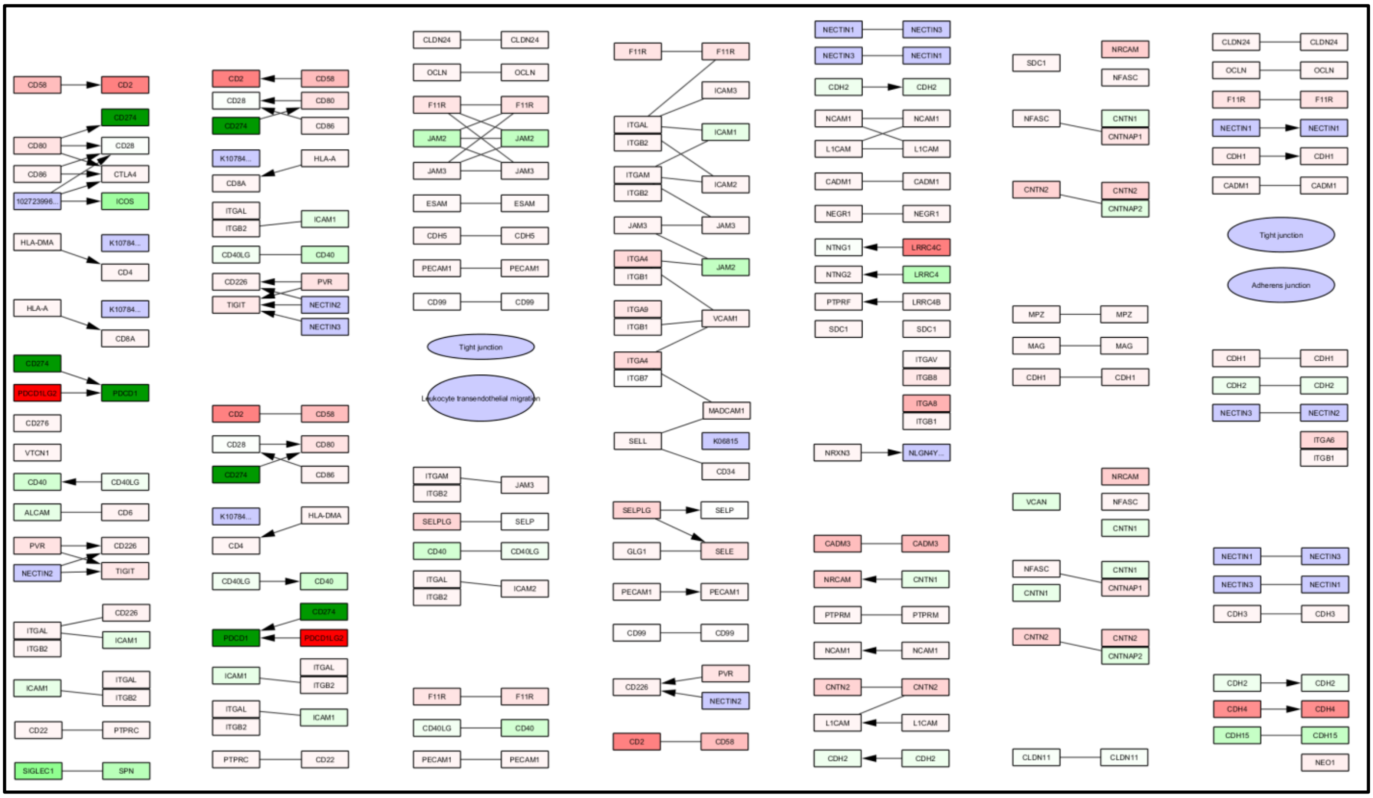

Suppl. Fig. S13 Takashima et al.

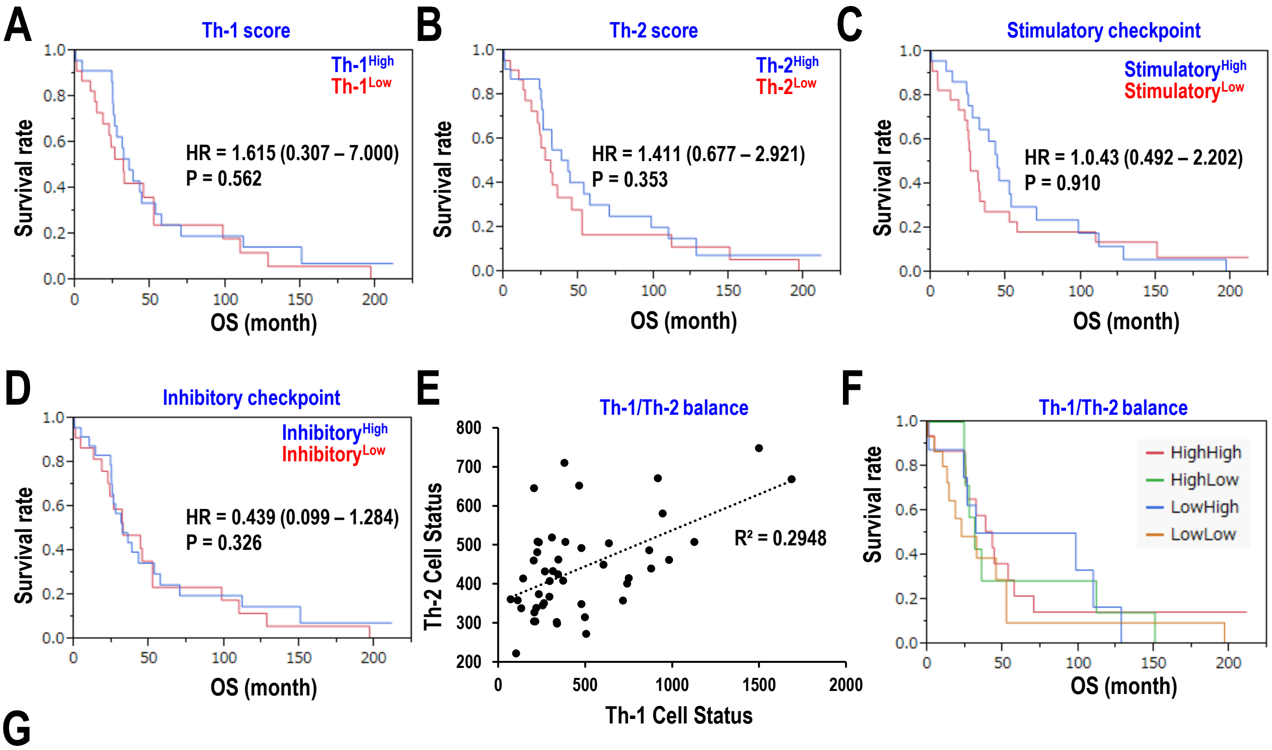

G

Subgroup	Th-1	Th-2	HR	95%CI	P-value
HighHigh	High	High	1.684	0.581 - 6.067	0.351
HighLow	High	Low	1.953	0.584 - 7.507	0.278
LowHigh	Low	High	1	-	-
LowLow	Low	Low	1.888	0.580 - 7.194	0.295

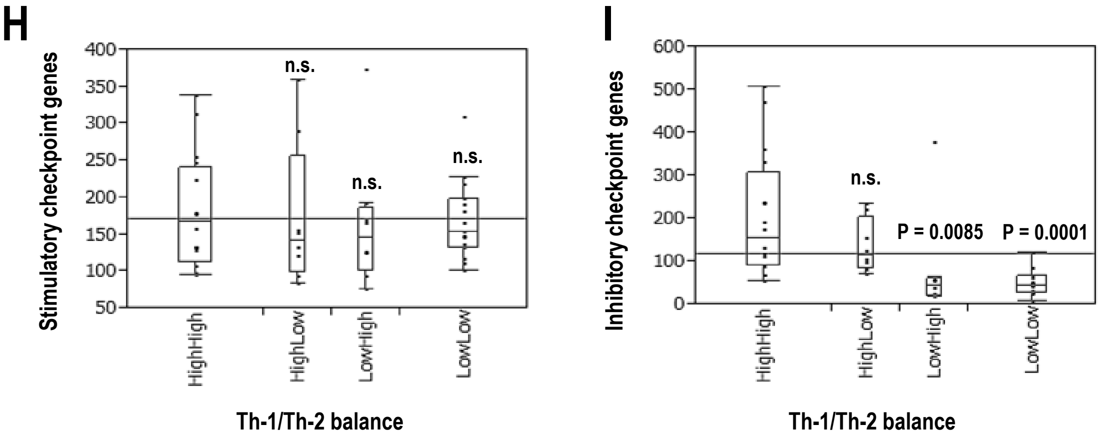

Suppl. Fig. S14

Takashima et al.

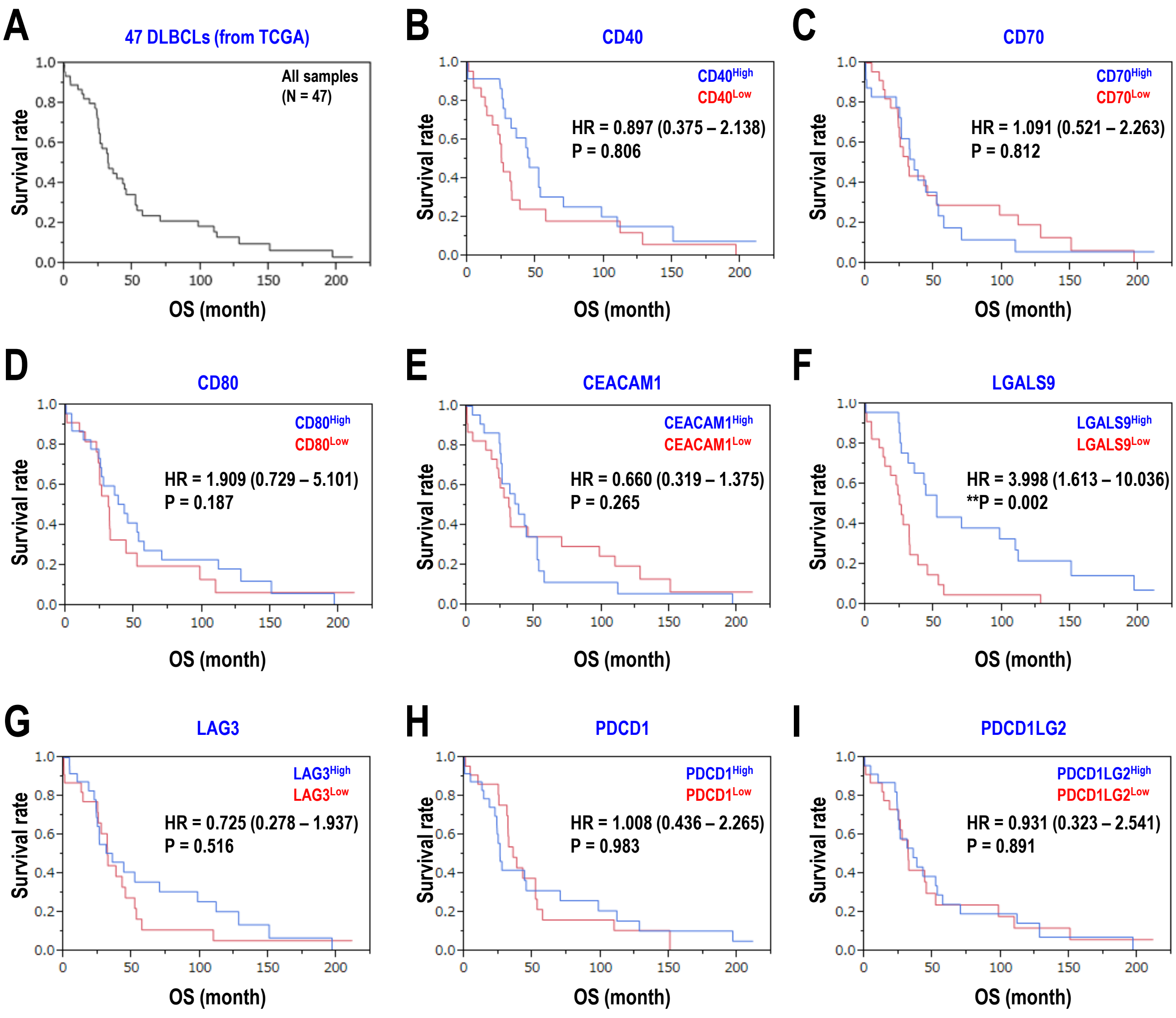

Suppl. Table S1. The genes related to Th-1 and Th-2 helper T cell status, and immune checkpoint.

Symbol	GeneBank	Gene ID	Description	Alias	Th-1 cells	Th-2 cells	Stimulatory checkpoint	Inhibitory checkpoint	References
CD28	NM_006139	940	CD28 molecule(CD28)	Tp44	CD28	CD28	CD28		59
CD3D	NM_000732	915	CD3d molecule(CD3D)	CD3-DELTA, IMD19, T3D	CD3D	CD3D			62, 92
CD3E	NM_000733	916	CD3e molecule(CD3E)	IMD18, T3E, TCRE	CD3E	CD3E			62, 92
CD3G	NM_000073	917	CD3g molecule(CD3G)	CD3-GAMMA, IMD17, T3G	CD3G	CD3G			62, 92
CD4	NM_000616	920	CD4 molecule(CD4)	CD4mut	CD4	CD4			53, 62, 65, 67, 69, 71, 80, 82, 83, 92
CD40LG	NM_000074	959	CD40 ligand(CD40LG)	CD154, CD40L, HIGM1, IGM, IMD3, T-BAM, TNFSF5, TRAP, gp39, hCD40L	CD40LG	CD40LG	CD40LG		83
CSF2	NM_000758	1437	colony stimulating factor 2(CSF2)	GMCSF	CSF2	CSF2			62, 96
IFNG	NM_000619	3458	interferon gamma(IFNG)	IFG, IFI	IFNG				63, 65, 69, 79, 80, 83, 90, 91
IL12RB2	NM_001559	3595	interleukin 12 receptor subunit beta 2(IL12RB2)		IL12RB2				51, 90
IL18R1	NM_003855	8809	interleukin 18 receptor 1(IL18R1)	CD218a, CDw218a, IL-1Rrp, IL18RA, IL1RRP	IL18R1	IL18R1			70,
IL2	NM_000586	3558	interleukin 2(IL2)	IL-2, TCGF, lymphokine	IL2				53, 80, 88
IL3	NM_000588	3562	interleukin 3(IL3)	IL-3, MCGF, MULTI-CSF	IL3	IL3			82
LTA	NM_000595	4049	lymphotoxin alpha(LTA)	LT, TNFB, TNFSF1	LTA				52
STAT1	NM_007315	6772	signal transducer and activator of transcription 1(STAT1)	CANDF7, IMD31A, IMD31B, IMD31C, ISGF-3, STAT91	STAT1				81
STAT4	NM_003151	6775	signal transducer and activator of transcription 4(STAT4)	SLEB11	STAT4				83, 95
TBX21	NM_013351	30009	T-box 21(TBX21)	T-PET, T-bet, TBET, TBLYM	TBX21				83, 90, 92
TNF	NM_000594	7124	tumor necrosis factor(TNF)	DIF, TNF-alpha, TNFA, TNFSF2	TNF				53
GATA3	NM_002051	2625	GATA binding protein 3(GATA3)	HDR, HDRS		GATA3			82, 83, 90
IL10	NM_000572	3586	interleukin 10(IL10)	CSIF, GVHDS, IL-10, IL10A, TGIF		IL10			69
IL13	NM_002188	3596	interleukin 13(IL13)	IL-13, P600		IL13			78, 82, 83
IL4	NM_000589	3565	interleukin 4(IL4)	BCGF-1, BCGF1, BSF-1, BSF1, IL-4		IL4			78, 82, 88, 90, 92
IL5	NM_000879	3567	interleukin 5(IL5)	EDF, IL-5, TRF		IL5			61, 82
IL6	NM_000600	3569	interleukin 6(IL6)	BSF2, HGF, HSF, IFNB2, IL-6		IL6			81, 83
IL9	NM_000590	3578	interleukin 9(IL9)	HP40, IL-9, P40		IL9			71, 88
STAT6	NM_003153	6778	signal transducer and activator of transcription 6(STAT6)	D12S1644, IL-4-STAT, STAT6B, STAT6C		STAT6			78
TGFB3	NM_003239	7043	transforming growth factor beta 3(TGFB3)	TGFB3, ARVD, ARVD1, RNHF, TGF-beta3, Transforming growth factor, beta 3, LDS5		TGFB3			85
CD226	NM_006566	10666	CD226 molecule(CD226)				CD226		59, 60
CD27	NM_001242	939	CD27 molecule(CD27)	S152, S152. LPFS2, T14, TNFRSF7, Tp55			CD27		80
CD276	NM_025240	80381	CD276 molecule(CD276)	4Ig-B7-H3, B7-H3, B7H3, B7RP-2			CD276	CD276	76
CD40	NM_152854	958	CD40 molecule(CD40)				CD40		51, 80
CD70	NM_001252	970	CD70 molecule(CD70)				CD70		54
CD80	NM_005191	941	CD80 molecule(CD80)				CD80	CD80	51
HHLA2	NM_007072	11148	HERV-H LTR-associating 2(HHLA2)				HHLA2		73
ICOS	NM_012092	29851	inducible T-cell costimulator(ICOS)	AILIM, CD278, CVID1			ICOS		67
IL2RB	NM_000878	3560	interleukin 2 receptor subunit beta(IL2RB)	CD122, IL15RB, P70-75			IL2RB		74
PVR	NM_006505	5817	poliovirus receptor(PVR)				PVR	PVR	89
TMIGD2	NM_144615	126259	transmembrane and immunoglobulin domain containing 2(TMIGD2)				TMIGD2		73
TNFRSF14	NM_003820	8764	TNF receptor superfamily member 14(TNFRSF14)	ATAR, CD270, HVEA, HVEM, LIGHTR, TR2			TNFRSF14	TNFRSF14	87
TNFRSF18	NM_004195	8784	TNF receptor superfamily member 18(TNFRSF18)	AITR, CD357, GITR, GITR-D			TNFRSF18		67, 75, 92
TNFRSF4	NM_003327	7293	TNF receptor superfamily member 4(TNFRSF4)				TNFRSF4		92
TNFRSF9	NM_001561	3604	TNF receptor superfamily member 9(TNFRSF9)	4-1BB, CD137, CDw137, ILA			TNFRSF9		92
TNFSF14	NM_003807	8740	tumor necrosis factor superfamily member 14(TNFSF14)				TNFSF14		58
TNFSF18	NM_005092	8995	tumor necrosis factor superfamily member 18(TNFSF18)				TNFSF18		75
TNFSF4	NM_003326	7292	tumor necrosis factor superfamily member 4(TNFSF4)	CD134L, CD252, GP34, OX-40L, OX4OL, TXGP1			TNFSF4		54, 67
TNFSF9	NM_003811	8744	tumor necrosis factor superfamily member 9(TNFSF9)				TNFSF9		86
BTLA	NM_181780	151888	B and T lymphocyte associated(BTLA)	BTLA1, CD272				BTLA	57
CD160	NM_007053	11126	CD160 molecule(CD160)					CD160	57
CD274	NM_014143	29126	CD274 molecule(CD274)	B7-H, B7H1, PD-L1, PDCD1L1, PDCD1LG1, PDL1				CD274	61, 66, 69, 72, 80, 84, 90, 94
CD86	NM_175862	942	CD86 molecule(CD86)					CD86	51
CD96	NM_198196	10225	CD96 molecule(CD96)					CD96	59, 68
CEACAM1	NM_001712	634	carcinoembryonic antigen related cell adhesion molecule 1(CEACAM1)					CEACAM1	56, 72
CTLA4	NM_005214	1493	cytotoxic T-lymphocyte associated protein 4(CTLA4)	ALPS5, CD, CD152, CELIAC3, CTLA-4, GRD4, GSE, IDDM12				CTLA4	59, 61, 66-68, 72, 84, 91, 94
HAVCR2	NM_032782	84868	hepatitis A virus cellular receptor 2(HAVCR2)	CD366, HAVcr-2, KIM-3, TIM3, TIMD-3, TIMD3, Tim-3				HAVCR2	72, 84
IDO1	NM_002164	3620	indoleamine 2,3-dioxygenase 1(IDO1)	IDO, IDO-1, INDO				IDO1	80
LAG3	NM_002286	3902	lymphocyte activating 3(LAG3)	CD223				LAG3	84
LGALS3	NM_002306	3958	galectin 3(LGALS3)					LGALS3	55, 64
LGALS9	NM_002308	3965	galectin 9(LGALS9)					LGALS9	55, 63
PDCD1	NM_005018	5133	programmed cell death 1(PDCD1)	CD279, PD-1, PD1, SLEB2, hPD-1, hPD-l, hSLE1				PDCD1	53, 59, 61, 66, 68, 69, 72, 84, 90, 91, 94
PDCD1LG2	NM_025239	80380	programmed cell death 1 ligand 2(PDCD1LG2)	PDCD1LG2, B7DC, Btdc, CD273, PD-L2, PDCD1L2, PDL2, bA574F11.2, programmed cell death 1 ligand 2				PDCD1LG2	90
TIGIT	NM_173799	201633	T-cell immunoreceptor with Ig and ITIM domains(TIGIT)					TIGIT	59, 60, 68, 84, 89
VSIR	NM_022153	64115	chromosome 10 open reading frame 54(C10orf54)	VISTA, B7-H5, B7H5, GI24, PP2135, SISP1, DD1alpha, C10orf54, PD-1H, V-set immunoregulatory receptor				VISTA	93
VTCN1	NM_024626	79679	V-set domain containing T cell activation inhibitor 1(VTCN1)					VTCN1	77

Supple. Table S2. The numbers of samples into the subgroups divided by the Th-1/2 status and the gene expression.

	Number (%)				
Correlation with checkpoint	highhigh (n = 6)	highlow (n = 17)	lowhigh (n = 3)	lowlow (n = 5)	p-value
(Stimulatory checkpoint molecules)					
CD40.001cs = low (%)	3 (50.0)	14 (82.4)	1 (33.3)	1 (20.0)	0.044
(Inhibitory checkpoint molecules)					
PDCD1LG2.201cs = low (%)	1 (16.7)	3 (17.6)	3 (100.0)	5 (100.0)	0.001
PDCD1.003cs = low (%)	1 (16.7)	13 (76.5)	3 (100.0)	1 (20.0)	0.008
PDCD1.002cs = low (%)	4 (66.7)	17 (100.0)	3 (100.0)	3 (60.0)	0.037
PDCD1LG2.001cs = low (%)	1 (16.7)	7 (41.2)	3 (100.0)	4 (80.0)	0.047

Suppl. Table S3. The expression of stimulatory checkpoint genes in the subgroups divided by the Th-1/2 status.

	mean (sd)				
Correlation with checkpoint	highhigh (n = 6)	highlow (n = 17)	lowhigh (n = 3)	lowlow (n = 5)	p-value
(Stimulatory checkpoint molecules)					
CD40-001	47.05 (28.98)	28.52 (13.34)	42.89 (19.65)	46.40 (21.19)	0.11
CD40-002	2.78 (3.13)	1.13 (1.33)	3.11 (0.42)	1.59 (2.22)	0.189
CD27-001	23.60 (24.84)	33.81 (22.95)	61.73 (59.08)	24.37 (11.84)	0.212
TNFRSF4-001	2.71 (4.36)	0.98 (1.07)	0.34 (0.24)	0.87 (1.40)	0.301
TNFRSF18-002	0.46 (1.06)	0.10 (0.17)	0.00 (0.00)	0.09 (0.16)	0.381
TNFRSF18-001	2.02 (3.59)	0.86 (1.28)	0.08 (0.10)	0.63 (0.81)	0.431
IL2RB-001	20.48 (8.56)	19.04 (16.52)	15.76 (17.06)	24.40 (18.81)	0.879
TNFRSF18-003	0.04 (0.09)	0.05 (0.10)	0.04 (0.04)	0.06 (0.09)	0.98

Suppl. Table S4. The expression of inhibitory checkpoint genes in the subgroups divided by the Th-1/2 status.

	mean (sd)				
**Correlation with checkpoint**	**highhigh (n = 6)**	**highlow (n = 17)**	**lowhigh (n = 3)**	**lowlow (n = 5)**	**p-value**
**(Inhibitory checkpoint molecules)**					
**IDO1-002**	2.27 (2.71)	2.09 (1.77)	0.13 (0.23)	0.18 (0.14)	0.089
**ADORA2A-001**	15.71 (7.11)	10.82 (6.66)	23.82 (18.21)	19.75 (16.68)	0.124
**PDCD1-003**	0.44 (0.30)	0.20 (0.29)	0.00 (0.00)	1.83 (3.48)	0.14
**PDCD1-001**	15.50 (13.01)	6.98 (7.03)	2.92 (3.70)	28.31 (44.21)	0.142
**LAG3-001**	8.46 (8.63)	6.36 (6.26)	2.30 (3.17)	2.22 (1.73)	0.297
**BTLA-001**	10.46 (5.57)	9.75 (9.00)	6.39 (4.12)	4.50 (4.83)	0.501
**BTLA-002**	5.97 (3.46)	4.08 (3.65)	2.94 (2.33)	3.26 (3.14)	0.512
**CD274-201**	0.59 (0.45)	0.71 (1.39)	0.30 (0.18)	0.00 (0.00)	0.622
**CD274-001**	13.30 (10.10)	13.69 (24.39)	5.17 (7.56)	2.55 (1.23)	0.656

Suppl. Table S5. The genes involved in the target pathway candidates of PCNSL.

Symbol	Description	Pathway ID	Pathway name	P value[1]	FDR[2]
(Gene)					
CD70	CD70 molecule	hsa04060	Cytokine-cytokine receptor interaction - Homo sapiens (human)	0.40714	1.26824E-06
PDCD1	programmed cell death 1	hsa04514	Cell adhesion molecules (CAMs) - Homo sapiens (human)	0.03052	7.07221E-06
PDCD1	programmed cell death 1	hsa04660	T cell receptor signaling pathway - Homo sapiens (human)	0.8122	7.07221E-06
(Isoform)					
CD70	CD70 molecule	hsa04060	Cytokine-cytokine receptor interaction - Homo sapiens (human)	0.00011	2.8291E-28
STAT1	signal transducer and activator of transcription 1	hsa04062	Chemokine signaling pathway - Homo sapiens (human)	0.05665	4.88683E-26
STAT1	signal transducer and activator of transcription 1	hsa04217	Necroptosis - Homo sapiens (human)	0.03025	4.88683E-26
STAT1	signal transducer and activator of transcription 1	hsa04380	Osteoclast differentiation - Homo sapiens (human)	0.06926	4.88683E-26
STAT1	signal transducer and activator of transcription 1	hsa04620	Toll-like receptor signaling pathway - Homo sapiens (human)	0.52897	4.88683E-26
STAT1	signal transducer and activator of transcription 1	hsa04621	NOD-like receptor signaling pathway - Homo sapiens (human)	0.19214	4.88683E-26
STAT1	signal transducer and activator of transcription 1	hsa04625	C-type lectin receptor signaling pathway - Homo sapiens (human)	0.08203	4.88683E-26
STAT1	signal transducer and activator of transcription 1	hsa04630	JAK-STAT signaling pathway - Homo sapiens (human)	0.36932	4.88683E-26
STAT1	signal transducer and activator of transcription 1	hsa04658	Th1 and Th2 cell differentiation - Homo sapiens (human)	0.39553	4.88683E-26
STAT1	signal transducer and activator of transcription 1	hsa04659	Th17 cell differentiation - Homo sapiens (human)	0.12082	4.88683E-26
STAT1	signal transducer and activator of transcription 1	hsa04917	Prolactin signaling pathway - Homo sapiens (human)	0.58677	4.88683E-26
STAT1	signal transducer and activator of transcription 1	hsa04919	Thyroid hormone signaling pathway - Homo sapiens (human)	0.11527	4.88683E-26
STAT1	signal transducer and activator of transcription 1	hsa04933	AGE-RAGE signaling pathway in diabetic complications - Homo sapiens (human)	0.0891	4.88683E-26
STAT1	signal transducer and activator of transcription 1	hsa05140	Leishmaniasis - Homo sapiens (human)	0.03315	4.88683E-26
STAT1	signal transducer and activator of transcription 1	hsa05145	Toxoplasmosis - Homo sapiens (human)	0.0555	4.88683E-26
STAT1	signal transducer and activator of transcription 1	hsa05152	Tuberculosis - Homo sapiens (human)	0.0116	4.88683E-26
STAT1	signal transducer and activator of transcription 1	hsa05160	Hepatitis C - Homo sapiens (human)	0.3057	4.88683E-26
STAT1	signal transducer and activator of transcription 1	hsa05161	Hepatitis B - Homo sapiens (human)	0.13629	4.88683E-26
STAT1	signal transducer and activator of transcription 1	hsa05162	Measles - Homo sapiens (human)	0.1347	4.88683E-26
STAT1	signal transducer and activator of transcription 1	hsa05164	Influenza A - Homo sapiens (human)	0.04659	4.88683E-26
STAT1	signal transducer and activator of transcription 1	hsa05165	Human papillomavirus infection - Homo sapiens (human)	0.47184	4.88683E-26
STAT1	signal transducer and activator of transcription 1	hsa05167	Kaposi sarcoma-associated herpesvirus infection - Homo sapiens (human)	0.01815	4.88683E-26
STAT1	signal transducer and activator of transcription 1	hsa05168	Herpes simplex virus 1 infection - Homo sapiens (human)	0.27506	4.88683E-26
STAT1	signal transducer and activator of transcription 1	hsa05169	Epstein-Barr virus infection - Homo sapiens (human)	0.00034	4.88683E-26
STAT1	signal transducer and activator of transcription 1	hsa05200	Pathways in cancer - Homo sapiens (human)	0.64897	4.88683E-26
STAT1	signal transducer and activator of transcription 1	hsa05212	Pancreatic cancer - Homo sapiens (human)	0.18234	4.88683E-26
STAT1	signal transducer and activator of transcription 1	hsa05321	Inflammatory bowel disease (IBD) - Homo sapiens (human)	0.65114	4.88683E-26
IL10	interleukin 10	hsa04060	Cytokine-cytokine receptor interaction - Homo sapiens (human)	0.00011	3.14171E-11
IL10	interleukin 10	hsa04068	FoxO signaling pathway - Homo sapiens (human)	0.06205	3.14171E-11
IL10	interleukin 10	hsa04625	C-type lectin receptor signaling pathway - Homo sapiens (human)	0.08203	3.14171E-11
IL10	interleukin 10	hsa04630	JAK-STAT signaling pathway - Homo sapiens (human)	0.36932	3.14171E-11
IL10	interleukin 10	hsa04660	T cell receptor signaling pathway - Homo sapiens (human)	0.02279	3.14171E-11
IL10	interleukin 10	hsa04672	Intestinal immune network for IgA production - Homo sapiens (human)	0.19988	3.14171E-11
IL10	interleukin 10	hsa05133	Pertussis - Homo sapiens (human)	0.67305	3.14171E-11
IL10	interleukin 10	hsa05140	Leishmaniasis - Homo sapiens (human)	0.03315	3.14171E-11
IL10	interleukin 10	hsa05142	Chagas disease (American trypanosomiasis) - Homo sapiens (human)	0.04537	3.14171E-11
IL10	interleukin 10	hsa05143	African trypanosomiasis - Homo sapiens (human)	0.87872	3.14171E-11
IL10	interleukin 10	hsa05144	Malaria - Homo sapiens (human)	0.24693	3.14171E-11
IL10	interleukin 10	hsa05145	Toxoplasmosis - Homo sapiens (human)	0.0555	3.14171E-11
IL10	interleukin 10	hsa05146	Amoebiasis - Homo sapiens (human)	0.92282	3.14171E-11
IL10	interleukin 10	hsa05150	Staphylococcus aureus infection - Homo sapiens (human)	0.50747	3.14171E-11
IL10	interleukin 10	hsa05152	Tuberculosis - Homo sapiens (human)	0.0116	3.14171E-11
IL10	interleukin 10	hsa05310	Asthma - Homo sapiens (human)	0.86801	3.14171E-11
IL10	interleukin 10	hsa05320	Autoimmune thyroid disease - Homo sapiens (human)	0.69623	3.14171E-11
IL10	interleukin 10	hsa05321	Inflammatory bowel disease (IBD) - Homo sapiens (human)	0.65114	3.14171E-11
IL10	interleukin 10	hsa05322	Systemic lupus erythematosus - Homo sapiens (human)	0.00056	3.14171E-11
IL10	interleukin 10	hsa05330	Allograft rejection - Homo sapiens (human)	0.38135	3.14171E-11
PDCD1	programmed cell death 1	hsa04514	Cell adhesion molecules (CAMs) - Homo sapiens (human)	0.58693	3.57908E-11
PDCD1	programmed cell death 1	hsa04660	T cell receptor signaling pathway - Homo sapiens (human)	0.02279	3.57908E-11
CD274	CD274 molecule	hsa04514	Cell adhesion molecules (CAMs) - Homo sapiens (human)	0.58693	3.74162E-08
STAT6	signal transducer and activator of transcription 6	hsa04217	Necroptosis - Homo sapiens (human)	0.03025	1.68264E-05
STAT6	signal transducer and activator of transcription 6	hsa04630	JAK-STAT signaling pathway - Homo sapiens (human)	0.36932	1.68264E-05
STAT6	signal transducer and activator of transcription 6	hsa04658	Th1 and Th2 cell differentiation - Homo sapiens (human)	0.39553	1.68264E-05
STAT6	signal transducer and activator of transcription 6	hsa04659	Th17 cell differentiation - Homo sapiens (human)	0.12082	1.68264E-05
STAT6	signal transducer and activator of transcription 6	hsa05161	Hepatitis B - Homo sapiens (human)	0.13629	1.68264E-05
STAT6	signal transducer and activator of transcription 6	hsa05200	Pathways in cancer - Homo sapiens (human)	0.64897	1.68264E-05
STAT6	signal transducer and activator of transcription 6	hsa05321	Inflammatory bowel disease (IBD) - Homo sapiens (human)	0.65114	1.68264E-05
CD3G	CD3g molecule	hsa04640	Hematopoietic cell lineage - Homo sapiens (human)	0.77392	4.99832E-05
CD3G	CD3g molecule	hsa04658	Th1 and Th2 cell differentiation - Homo sapiens (human)	0.39553	4.99832E-05
CD3G	CD3g molecule	hsa04659	Th17 cell differentiation - Homo sapiens (human)	0.12082	4.99832E-05
CD3G	CD3g molecule	hsa04660	T cell receptor signaling pathway - Homo sapiens (human)	0.02279	4.99832E-05
CD3G	CD3g molecule	hsa05142	Chagas disease (American trypanosomiasis) - Homo sapiens (human)	0.04537	4.99832E-05
CD3G	CD3g molecule	hsa05162	Measles - Homo sapiens (human)	0.1347	4.99832E-05
CD3G	CD3g molecule	hsa05166	Human T-cell leukemia virus 1 infection - Homo sapiens (human)	0.00016	4.99832E-05
CD3G	CD3g molecule	hsa05169	Epstein-Barr virus infection - Homo sapiens (human)	0.00034	4.99832E-05
CD3G	CD3g molecule	hsa05170	Human immunodeficiency virus 1 infection - Homo sapiens (human)	0.00637	4.99832E-05
TNFRSF14	TNF receptor superfamily member 14	hsa04060	Cytokine-cytokine receptor interaction - Homo sapiens (human)	0.00011	0.003353038
TNFRSF14	TNF receptor superfamily member 14	hsa05168	Herpes simplex virus 1 infection - Homo sapiens (human)	0.27506	0.003353038
CD40	CD40 molecule	hsa04060	Cytokine-cytokine receptor interaction - Homo sapiens (human)	0.00011	0.005815722
CD40	CD40 molecule	hsa04064	NF-kappa B signaling pathway - Homo sapiens (human)	0.39553	0.005815722
CD40	CD40 molecule	hsa04514	Cell adhesion molecules (CAMs) - Homo sapiens (human)	0.58693	0.005815722
CD40	CD40 molecule	hsa04620	Toll-like receptor signaling pathway - Homo sapiens (human)	0.52897	0.005815722
CD40	CD40 molecule	hsa04672	Intestinal immune network for IgA production - Homo sapiens (human)	0.19988	0.005815722
CD40	CD40 molecule	hsa05144	Malaria - Homo sapiens (human)	0.24693	0.005815722
CD40	CD40 molecule	hsa05145	Toxoplasmosis - Homo sapiens (human)	0.0555	0.005815722
CD40	CD40 molecule	hsa05166	Human T-cell leukemia virus 1 infection - Homo sapiens (human)	0.00016	0.005815722
CD40	CD40 molecule	hsa05169	Epstein-Barr virus infection - Homo sapiens (human)	0.00034	0.005815722
CD40	CD40 molecule	hsa05202	Transcriptional misregulation in cancer - Homo sapiens (human)	0.3436	0.005815722

CD40	CD40 molecule	hsa05310	Asthma - Homo sapiens (human)	0.86801	0.005815722
CD40	CD40 molecule	hsa05320	Autoimmune thyroid disease - Homo sapiens (human)	0.69623	0.005815722
CD40	CD40 molecule	hsa05322	Systemic lupus erythematosus - Homo sapiens (human)	0.00056	0.005815722
CD40	CD40 molecule	hsa05330	Allograft rejection - Homo sapiens (human)	0.38135	0.005815722
CD40	CD40 molecule	hsa05340	Primary immunodeficiency - Homo sapiens (human)	0.24289	0.005815722
CD40	CD40 molecule	hsa05416	Viral myocarditis - Homo sapiens (human)	0.05372	0.005815722
IL6	interleukin 6	hsa04060	Cytokine-cytokine receptor interaction - Homo sapiens (human)	0.00011	0.009267485
IL6	interleukin 6	hsa04066	HIF-1 signaling pathway - Homo sapiens (human)	0.04139	0.009267485
IL6	interleukin 6	hsa04068	FoxO signaling pathway - Homo sapiens (human)	0.06205	0.009267485
IL6	interleukin 6	hsa04151	PI3K-Akt signaling pathway - Homo sapiens (human)	0.91967	0.009267485
IL6	interleukin 6	hsa04218	Cellular senescence - Homo sapiens (human)	0.00089	0.009267485
IL6	interleukin 6	hsa04620	Toll-like receptor signaling pathway - Homo sapiens (human)	0.52897	0.009267485
IL6	interleukin 6	hsa04621	NOD-like receptor signaling pathway - Homo sapiens (human)	0.19214	0.009267485
IL6	interleukin 6	hsa04623	Cytosolic DNA-sensing pathway - Homo sapiens (human)	1	0.009267485
IL6	interleukin 6	hsa04625	C-type lectin receptor signaling pathway - Homo sapiens (human)	0.08203	0.009267485
IL6	interleukin 6	hsa04630	JAK-STAT signaling pathway - Homo sapiens (human)	0.36932	0.009267485
IL6	interleukin 6	hsa04640	Hematopoietic cell lineage - Homo sapiens (human)	0.77392	0.009267485
IL6	interleukin 6	hsa04657	IL-17 signaling pathway - Homo sapiens (human)	0.92318	0.009267485
IL6	interleukin 6	hsa04659	Th17 cell differentiation - Homo sapiens (human)	0.12082	0.009267485
IL6	interleukin 6	hsa04668	TNF signaling pathway - Homo sapiens (human)	0.66209	0.009267485
IL6	interleukin 6	hsa04672	Intestinal immune network for IgA production - Homo sapiens (human)	0.19988	0.009267485
IL6	interleukin 6	hsa04931	Insulin resistance - Homo sapiens (human)	0.78843	0.009267485
IL6	interleukin 6	hsa04932	Non-alcoholic fatty liver disease (NAFLD) - Homo sapiens (human)	0.00355	0.009267485
IL6	interleukin 6	hsa04933	AGE-RAGE signaling pathway in diabetic complications - Homo sapiens (human)	0.0891	0.009267485
IL6	interleukin 6	hsa05020	Prion diseases - Homo sapiens (human)	0.29813	0.009267485
IL6	interleukin 6	hsa05132	Salmonella infection - Homo sapiens (human)	0.17399	0.009267485
IL6	interleukin 6	hsa05133	Pertussis - Homo sapiens (human)	0.67305	0.009267485
IL6	interleukin 6	hsa05134	Legionellosis - Homo sapiens (human)	0.2767	0.009267485
IL6	interleukin 6	hsa05142	Chagas disease (American trypanosomiasis) - Homo sapiens (human)	0.04537	0.009267485
IL6	interleukin 6	hsa05143	African trypanosomiasis - Homo sapiens (human)	0.87872	0.009267485
IL6	interleukin 6	hsa05144	Malaria - Homo sapiens (human)	0.24693	0.009267485
IL6	interleukin 6	hsa05146	Amoebiasis - Homo sapiens (human)	0.92282	0.009267485
IL6	interleukin 6	hsa05152	Tuberculosis - Homo sapiens (human)	0.0116	0.009267485
IL6	interleukin 6	hsa05161	Hepatitis B - Homo sapiens (human)	0.13629	0.009267485
IL6	interleukin 6	hsa05162	Measles - Homo sapiens (human)	0.1347	0.009267485
IL6	interleukin 6	hsa05163	Human cytomegalovirus infection - Homo sapiens (human)	0.03484	0.009267485
IL6	interleukin 6	hsa05164	Influenza A - Homo sapiens (human)	0.04659	0.009267485
IL6	interleukin 6	hsa05166	Human T-cell leukemia virus 1 infection - Homo sapiens (human)	0.00016	0.009267485
IL6	interleukin 6	hsa05167	Kaposi sarcoma-associated herpesvirus infection - Homo sapiens (human)	0.01815	0.009267485
IL6	interleukin 6	hsa05168	Herpes simplex virus 1 infection - Homo sapiens (human)	0.27506	0.009267485
IL6	interleukin 6	hsa05169	Epstein-Barr virus infection - Homo sapiens (human)	0.00034	0.009267485
IL6	interleukin 6	hsa05200	Pathways in cancer - Homo sapiens (human)	0.64897	0.009267485
IL6	interleukin 6	hsa05202	Transcriptional misregulation in cancer - Homo sapiens (human)	0.3436	0.009267485
IL6	interleukin 6	hsa05321	Inflammatory bowel disease (IBD) - Homo sapiens (human)	0.65114	0.009267485
IL6	interleukin 6	hsa05323	Rheumatoid arthritis - Homo sapiens (human)	0.18565	0.009267485
IL6	interleukin 6	hsa05332	Graft-versus-host disease - Homo sapiens (human)	0.48047	0.009267485
IL6	interleukin 6	hsa05410	Hypertrophic cardiomyopathy (HCM) - Homo sapiens (human)	0.40843	0.009267485

NOTE: [1]P value was calculated in GSEA, [2]FDR; false discovery rate was calculated in edgeR.
